# Biotin-dependent metabolism controls germinal center fate and vaccine-induced antibody diversification

**DOI:** 10.1016/j.isci.2026.117999

**Published:** 2026-10-21

**Authors:** Chu-Han Hsieh, Wilson Aala, Ying-Chen Tsai, Ya-Fang Huang, Miao-Shan Lin, Daw-Yang Hwang, Dar-Bin Shieh, Hsiang-Hsuan Sung, Si-Tse Jiang, Ching-Wei Lin, Chih-An Chen, Michael Warren Hughes, Peng-Chieh Chen, Chi-Chang Shieh

**Affiliations:** 1 Institute of Clinical Medicine, College of Medicine, National Cheng-Kung University, Tainan, Taiwan; 2 Research Center of Clinical Medicine, College of Medicine, National Cheng Kung University Hospital, National Cheng-Kung University, Tainan, Taiwan; 3 Department of Pediatrics, National Cheng Kung University Hospital, Tainan, Taiwan; 4 National Center for Biomodels, National Institutes of Applied Reasearch, Tainan, Taiwan; 5 National Institute of Cancer Research, National Health Research Institutes, Tainan, Taiwan; 6 Institute of Oral Medicine, College of Medicine, National Cheng-Kung University, Tainan, Taiwan; 7 L’Istituto di Ricerca Scientifica Fondazione Human Technopole, Milan, Italy; 8 National Center for Biomodels, National Institutes of Applied Research, Taipei, Taiwan; 9 International Research Center for Wound Repair and Regeneration, National Cheng Kung University, Tainan, Taiwan; 10 Department of Life Science, College of Bioscience and Biotechnology, National Cheng Kung University, Tainan, Taiwan

**Keywords:** B cell, germinal center, metabolic rewiring, vaccine response, biotin

## Abstract

Nutrient availability shapes antibody responses, yet its role in metabolic checkpoints governing germinal center (GC) B-cell differentiation remains incompletely defined. Using heterozygous (HZ) *Slc5a6* M60K mice modeling human loss-of-function mutations in the multivitamin transporter SLC5A6, we found that deficient biotin imports impaired mitochondrial metabolism and restricted preGC-to-dark zone progression after vaccination. Single-cell transcriptomics and RNA velocity revealed disrupted GC trajectories, while B-cell receptor repertoire analysis showed impaired class-switch recombination, somatic hypermutation, and vaccine-induced clonal diversity. Spatial multiplexed imaging showed reduced GL7-high GC-associated B-cell regions. Flow cytometric analyses revealed reduced frequencies of RBD-specific B cells and RBD-specific GL7^+^CD95^+^ GC B cells in HZ mice, with higher frequencies after biotin supplementation. Pseudovirus assays demonstrated impaired neutralization of Wuhan-Hu-1 and Omicron BA.2. Dietary biotin supplementation restored GC-associated differentiation, antibody diversification, and vaccine-induced humoral responses. These findings identify biotin-dependent mitochondrial metabolism as an important checkpoint linking micronutrient availability to GC B-cell differentiation and vaccine responsiveness.

## Highlights

• Biotin import regulates germinal center B-cell differentiation and humoral immunity
• Impaired biotin import restricts preGC-to-cycling DZ-like GC progression
• Biotin rescues antibody diversification and vaccine-induced humoral responses

## Graphical abstract

**Figure:**
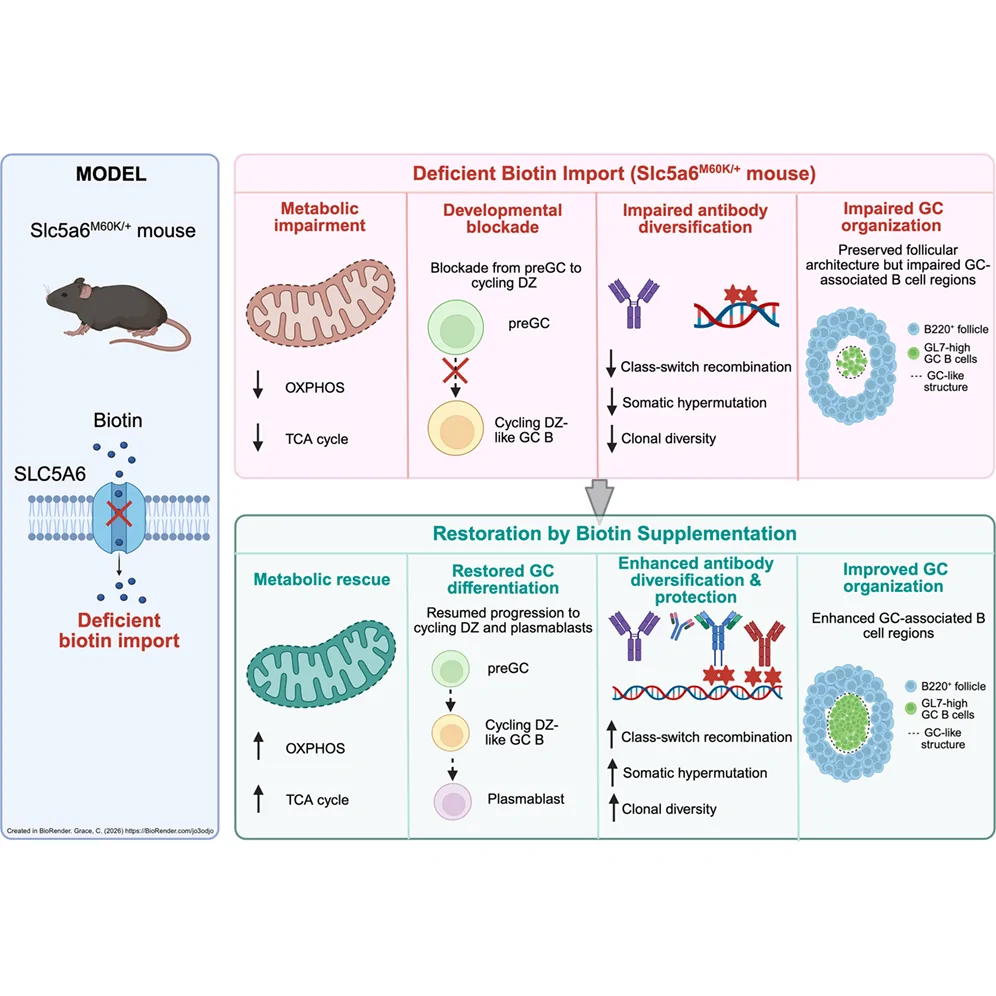

## Teaser

Biological sciences; Molecular biology; Immunology; Cell biology

## Introduction

Metabolic reprogramming has been revealed to be critical for immune cell activation, proliferation, and differentiation.^1^^,^^2^ Lymphocytes need to undergo dynamic shifts in metabolic pathways to support their distinct functional states. Naive and resting memory lymphocytes primarily utilize oxidative phosphorylation (OXPHOS) to meet their basal metabolic needs, while activated cells switch to aerobic glycolysis to accommodate the new demand for higher biosynthesis and ATP production.^3^^,^^4^ T cells exemplify this metabolic flexibility, with effector T cells relying on glycolysis and memory T cells reverting to OXPHOS and fatty acid oxidation.^5^^,^^6^^,^^7^^,^^8^ Similarly, macrophage subsets exhibit distinct metabolic programs aligned with their pro- or anti-inflammatory functions.^9^^,^^10^^,^^11^

B cell metabolism is uniquely complexed due to the dynamic functional maturation of B cells as they interact with antigens and other immune cells during development, activation, and differentiation.^12^^,^^13^ While B cell activation through the B cell receptor (BCR) enhances glucose uptake and glycolysis,^14^ metabolic programs in B cells are highly context dependent. Naive and early activated or pre-germinal center (preGC) B cells rely on glycolysis to support rapid proliferation, whereas germinal center (GC) B cells adopt a distinct oxidative metabolic state as they cycle between the dark and light zones. GC B cells generate the majority of their ATP through OXPHOS rather than aerobic glycolysis and preferentially utilize fatty-acid oxidation as a major energy source.^15^^,^^16^^,^^17^ Importantly, glucose in GC B cells is not primarily used for ATP production but is diverted into biosynthetic pathways, including the pentose phosphate pathway and one-carbon metabolism, providing substrates for nucleotide synthesis, redox balance, and anabolic growth. GC B cells also increase exogenous amino acid uptake and are sensitive to nutrient limitation, highlighting that GC responses are tightly coupled to environmental metabolic availability,^18^ thereby enabling affinity maturation and plasma cell differentiation. The GC reaction is crucial for generating high-affinity antibodies and long-lived memory B cells,^19^^,^^20^ with distinct metabolic profiles observed between dark zone (DZ) and light zone (LZ) B cells. By contrast, extrafollicular (EF) B cells which produce plasmablasts and antibodies with lower affinity rapidly, providing a faster yet less durable immune response,^21^ may have different metabolic programs. Despite these recent insights, the metabolic requirements of distinct B cell subsets, particularly in the context of vaccination, remain incompletely understood.

Biotin (vitamin B7) serves as an essential coenzyme for five carboxylases that catalyze key reactions involved in tricarboxylic acid (TCA) cycle, fatty acid synthesis, gluconeogenesis, and branched-chain amino acid catabolism. Its availability directly influences mitochondrial metabolism, a critical aspect for rapidly dividing cells. Biotin deficiency has been shown to impair mitochondrial structure and function, leading to reduced TCA cycle flux, diminished electron transport chain activity, and decreased OXPHOS.^22^ Cellular uptake of biotin is mediated primarily by SLC5A6, a sodium-dependent multivitamin transporter that also facilitates the uptake of pantothenic acid (vitamin B5) and lipoate. Mutations in the gene were associated with biotin deficiency and immune dysfunction in both humans and mouse models.^23^ In this study, we performed single-cell RNA sequencing (scRNA-seq) of splenic B cells from *Slc5a6* M60K heterozygous (HZ) mice, with or without dietary biotin supplementation, and compared them to wild-type (WT) controls under steady-state and post-vaccination conditions. Our findings reveal a previously unrecognized role for biotin in supporting GC B cell differentiation, clonal expansion, and vaccine-induced antibody responses. In this study, we identified a developmental blockade at the preGC stage associated with mitochondrial dysfunction and altered metabolic programming, which is reversible upon biotin replenishment. These results uncover critical metabolic vulnerabilities in B-cell development and highlight biotin as a potential modulator of humoral immunity.

## Results

### Biotin deficiency impairs mitochondrial respiration and selectively alters B-cell subset composition

To investigate how biotin-dependent metabolic processes regulate B cell development and function, we employed a gene-edited mouse model (*Slc5a6* M60K) harboring a loss-of-function mutation in the multivitamin transporter SLC5A6 (Figures S1A and S1B). This mutation corresponds to a human variant associated with primary B cell immunodeficiency, providing an *in vivo* model of functional biotin deficiency.^23^ Using the Seahorse XF assay, we assessed mitochondrial respiration in splenic B cells stimulated with F(ab′)_2_ anti-IgG/IgM and IL-4 to model an early activation/preGC state. Consistent with our previous observation that *Slc5a6* deficiency impairs mitochondrial respiration in activated naive B cells stimulated with IL-4 and IL-21,^23^ *Slc5a6* mutant (HZ) B cells exhibited significantly reduced oxygen consumption rates compared with WT controls under these activation conditions (Figure 1A). Both basal respiration and maximal respiratory capacity were markedly diminished in mutant cells (Figure 1B). Proton leak was also reduced (Figure 1B), suggesting globally suppressed mitochondrial flux rather than isolated uncoupling defects. Together, these findings indicate impaired OXPHOS and limited respiratory capacity in biotin-deficient B cells during activation. We next examined how biotin deficiency affects B cell maturation. Quantitative flow cytometric analysis revealed a significant reduction in both the absolute number and frequency of splenic follicular B cells in mutant mice, accompanied by a corresponding decrease in GC B-cell abundance (Figure 1C, left images; gating strategy shown in Figures S2A and S2B). In contrast, although marginal zone (MZ) B-cell numbers were slightly higher in mutant mice, their frequency within the splenic B-cell compartment was not significantly altered. Early splenic B-cell developmental subsets, including transitional T1, T2, and T3 B cells, as well as CD5^+^ B1 cells, were present at comparable absolute numbers and frequencies between WT and mutant mice (Figure S2C), indicating that early peripheral B cell development is largely preserved. To further assess the bone marrow precursor population that gives rise to the peripheral B2-cell compartment, we quantified immature B cells (B220^+^CD93^+^IgM^+^IgD^−^). No significant differences in either absolute numbers or frequencies were observed among WT, HZ, and HZ_b7 mice (Figures S3A and S3B), indicating that early bone marrow B cell development and generation of the peripheral B2-cell pool remain largely intact despite *Slc5a6* deficiency. In contrast, plasma cells quantified using a validated four-parameter gating strategy^24^ were significantly reduced in both absolute numbers and frequencies in mutant mice (Figure 1C, right images; gating strategy shown in Figure S3C). Plasma cell frequencies among total bone marrow cells were reduced from approximately ∼1% in controls to ∼0.6% in mutants, indicating impaired plasma cell generation and/or maintenance. Together, these findings indicate that biotin deficiency largely preserves early bone marrow B cell development while preferentially impairing peripheral follicular B-cell maturation, GC differentiation, and plasma cell generation.

**Figure 1:**
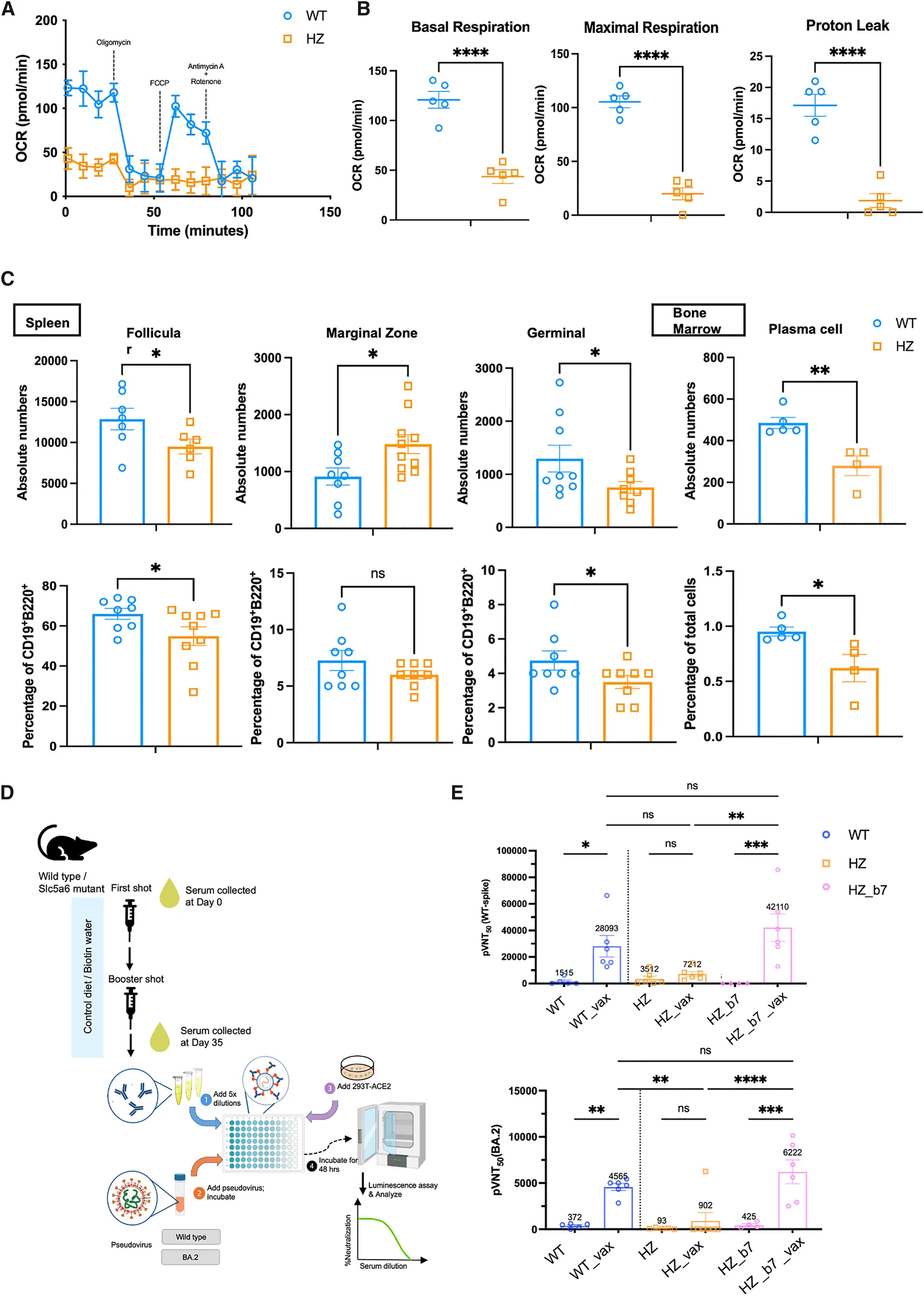
Biotin deficiency impairs B cell metabolism, subset composition and neutralizing antibody responses, restored by supplementation WT, wild-type mice; HZ, *Slc5a6* heterozygous mutant mice; HZ_b7, biotin-supplemented HZ mice. (A) Oxygen consumption rate (OCR) of total splenic B cells from WT and HZ mice measured using a Seahorse XF mitochondrial stress assay (*n* = 3 biological replicates per group, with each biological replicate representing an individual mouse). (B) Quantification of basal respiration, maximal respiration, and proton leak derived from OCR measurements (*n* = 3 biological replicates per group; five technical replicate wells per biological replicate). (C) Flow cytometric analysis of splenic and bone marrow B cell subsets (n = 6–10 mice). In the spleen (left), upper images show absolute cell numbers and lower images show frequencies within CD19^+^B220^+^ B cells. In the bone marrow (right), upper images show absolute plasma cell numbers and lower images show frequencies within total bone marrow cells. Data are presented as mean ± SEM with individual biological replicates indicated. (D) Schematic of the pseudovirus neutralization assay. Serum collected from mice on days 0 and 35 post-immunization was diluted (1:5), incubated with either Wuhan-Hu-1 (WT) or Omicron BA.2 SARS-CoV-2 pseudovirus for 1 h, and added to 293T-hACE2 cells. Infection levels were quantified by luminescence-based detection of viral entry. (E) Neutralizing antibody titers against SARS-CoV-2 WT and BA.2 variants measured before and after booster immunization (n = 4–6 mice per group). Data are presented as mean ± SEM. Statistical significance in (A–C) was determined using unpaired *t* tests. Statistical comparisons in (E) were performed using two-way ANOVA with multiple-comparisons correction. ∗*p* < 0.05; ∗∗*p* < 0.01; ∗∗∗*p* < 0.001; ∗∗∗∗*p* < 0.0001; ns, not significant.

### Biotin supplementation restores vaccine-induced neutralizing and cross-reactive antibody responses

We next assessed whether biotin supplementation could restore functional humoral immunity. WT, HZ, and biotin-supplemented mutant (HZ_b7) mice were immunized with a SARS-CoV-2 spike protein vaccine (based on the ancestral Wuhan-Hu-1 strain DNA sequence), and serum neutralizing antibody (NAb) titers were quantified two weeks after the booster using a pseudovirus-based neutralization assay (Figure 1D), a widely used surrogate for evaluating neutralizing activity.^25^ HZ mice exhibited markedly reduced NAb titers against both the homologous Wuhan-Hu-1 strain (Figure 1E, upper image) and the Omicron BA.2 variant (Figure 1E, lower image), indicating impaired vaccine-induced neutralizing responses across viral variants. Similarly, enzyme-linked immunosorbent assay (ELISA) demonstrated markedly reduced SARS-CoV-2 spike-specific total antibody responses in HZ mice, whereas biotin supplementation restored antigen-specific antibody production (Figure S3D). To further assess the antigen-specific B-cell responses, we performed flow cytometric analyses of SARS-CoV-2 RBD-binding B cells using dual-color fluorescent RBD probes. While an unvaccinated control showed only minimal background RBD dual-probe staining (Figure S3E), HZ mice showed significantly reduced frequencies of total RBD-specific B cells and RBD-specific GL7^+^CD95^+^ GC B cells compared with WT mice (Figures S3F and S3G), reflecting a lower representation of RBD-specific cells within the overall B-cell and GC compartments. Biotin-supplemented HZ_b7 mice showed higher frequencies of these populations, with values approaching those observed in WT mice. RBD-specific IgG1^+^ B cells, representing an antigen-specific class-switched B-cell population, also showed a lower frequency in HZ mice compared with WT mice and were present at higher frequencies in HZ_b7 mice (Figure S3H). A similar pattern was observed within the CD19^low/int^ CD138^hi^ plasmablast-like population, with fewer RBD-specific cells in HZ mice compared with WT mice and higher frequencies in HZ_b7 mice (Figure S3I). Together, these findings support an association between reduced biotin availability and lower vaccine-induced antigen-specific B-cell responses across multiple B-cell compartments, including GC-associated, IgG1 class-switched, and CD19^low/int^ CD138^hi^ plasmablast-like populations. Importantly, biotin supplementation restored NAb titers in HZ mice to levels comparable to WT for both viral strains, supporting a role for adequate biotin availability in the generation of NAb responses with cross-variant reactivity.

### Single-cell transcriptomics reveals biotin-sensitive alterations in B cell subsets and plasmablast differentiation

To define how biotin availability and *Slc5a6* deficiency affect B-cell heterogeneity and developmental states, we performed scRNA-seq on splenic B-lineage cells isolated from WT, HZ, and HZ_b7 mice, with or without SARS-CoV-2 spike protein vaccination (Figure 2A). Given the impaired mitochondrial respiration observed by Seahorse analysis, we next examined whether transcriptional programs associated with mitochondrial homeostasis were altered. Representative genes involved in mitophagy, mitochondrial biogenesis, and mitochondrial DNA replication showed no coordinated differences among experimental groups (Figure S4A), suggesting that the impaired OXPHOS is unlikely to result from widespread transcriptional dysregulation of mitochondrial turnover or biogenesis. To ensure dataset robustness and accurate cell-type assignment, transcript-level quality-control metrics and canonical marker expression were systematically evaluated. All clusters exhibited comparable mitochondrial read fractions and gene counts (Figures S4B–S4D). Uniform manifold approximation and projection (UMAP) overlays demonstrated broad expression of core B-lineage markers (*Cd19*, *Cd79a*, *Cd79b*, *Pax5*, and *Ms4a1*) across all clusters (Figures 2C, S4E, and S4F), confirming B-cell lineage identity. Unbiased clustering of high-quality transcriptomes identified 15 transcriptionally distinct B-cell populations (Figure 2B), and visualization of UMAP embeddings separated by genotype is shown in Figure S4G. To further confirm the hematopoietic origin of the analyzed cells and exclude potential stromal cell contamination, we examined expression of the pan-leukocyte marker *Ptprc* (CD45). As expected, *Ptprc* was broadly expressed across all B-lineage clusters without selective enrichment in any specific subset (Figure S4H). Cluster identities were subsequently assigned based on the integrated expression of an expanded panel of canonical follicular, GC, activation, proliferation, and memory-associated markers (Figures S5A and S5B), enabling annotation of transcriptionally distinct B-cell states.

**Figure 2:**
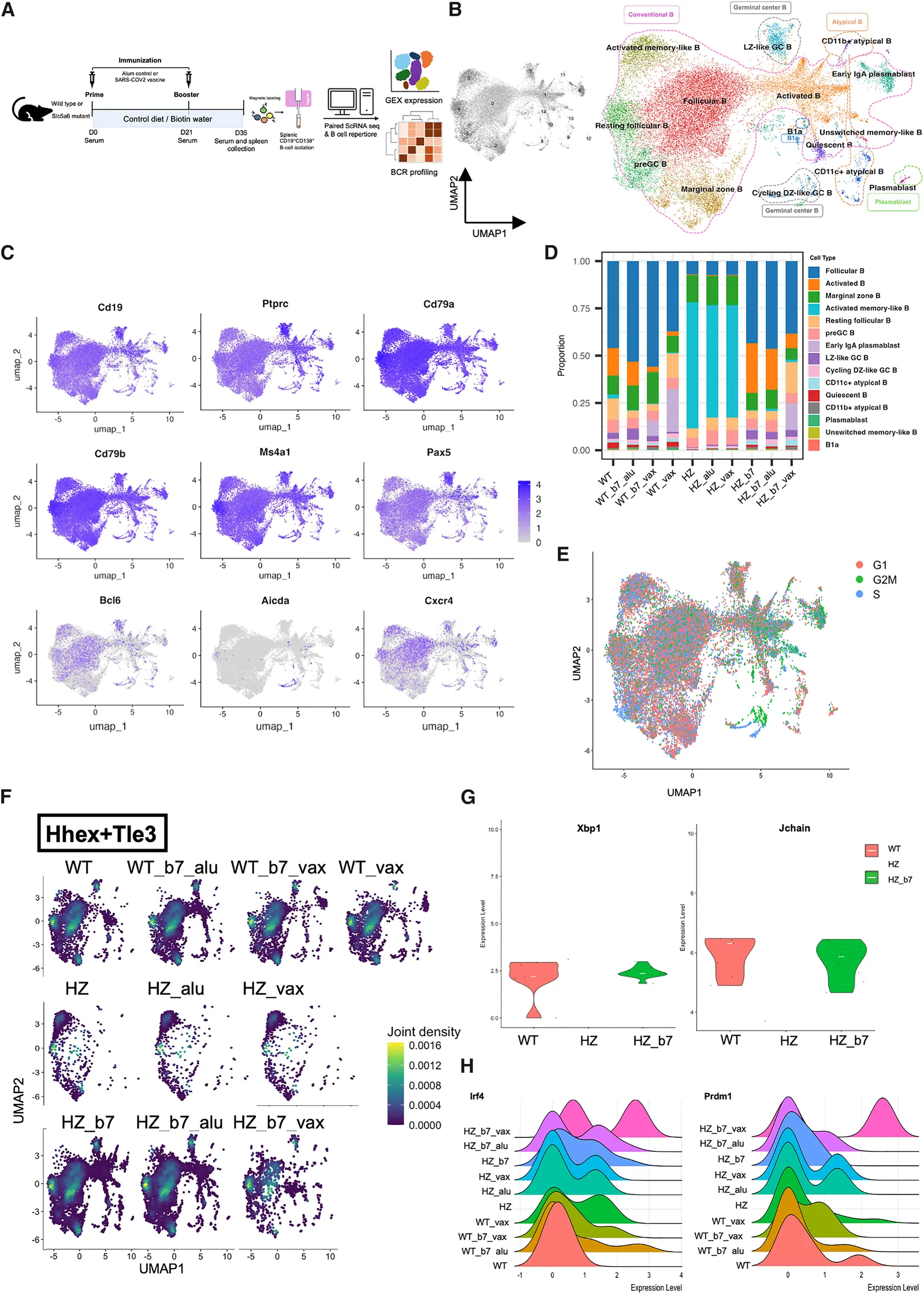
Single-cell transcriptomics delineates biotin-sensitive B cell subset alterations and plasmablast differentiation defects WT, wild-type mice; HZ, *Slc5a6* heterozygous mutant mice; HZ_b7, biotin-supplemented HZ mice. Suffixes “_alu” and “_vax” denote alum-only and SARS-CoV-2 vaccine-treated groups, respectively. (A) Experimental design. WT, HZ, and HZ_b7 mice (6–8 weeks old; *n* = 3–4 mice per condition) were assigned to untreated, alum-only, or SARS-CoV-2 vaccine groups. Splenic B cells from 3–4 mice were pooled to generate one biological sample per experimental condition for single-cell sequencing. Immunized mice received a prime injection on day 0 and a booster injection on day 21. Splenic CD19^+^CD138^+^ B-lineage cells were isolated on day 35 for droplet-based scRNA-seq and paired BCR repertoire analysis. (B) UMAP projection of 15 transcriptionally distinct B-cell clusters identified from the integrated scRNA-seq dataset. (C) UMAP visualization of canonical B-lineage and germinal center-associated marker expression used for cluster annotation. Uniform expression of core B-cell markers (*Cd19*, *Ptprc*, *Cd79a*, *Cd79b*, *Ms4a1*, and *Pax5*) confirmed B-lineage identity. Germinal center-associated populations were defined by enrichment of *Bcl6* and *Aicda*, whereas *Cxcr4* marked the cycling DZ-like GC B-cell population. (D) Proportional distribution of annotated B-cell populations across untreated, alum-only, and SARS-CoV-2 vaccine groups for each genotype. (E) Cell-cycle phase distribution (G1, S, and G2/M) across annotated B-cell populations. (F) Joint expression density of Hhex and Tle3 across all experimental groups visualized using Nebulosa. Density was calculated from normalized expression values in the integrated dataset and projected onto a shared UMAP embedding with panels split by condition. All images represent identical UMAP coordinates; differences therefore reflect changes in cell density rather than alterations in the embedding. (G) Violin plots showed normalized expression of the plasmablast-associated genes *Xbp1* and *Jchain* in WT, HZ, and HZ_b7 mice. (H) Density plots normalized expression distributions of *Irf4* and *Prdm1* across all experimental conditions.

Cluster annotation was guided by established marker gene expression and integration with published murine B cell reference datasets.^26^^,^^27^ Resting follicular B cells (C4) exhibited relatively high expression of *Cd24a* and *Hhex*,^28^ whereas follicular B cells (C0) showed relatively high expression of mature follicular markers including *Cxcr5*, Cd55, and *Fcer2a*. Together, these expression patterns suggest that C0 and C4 represent transcriptionally distinct states within the mature follicular B-cell compartment (Figure S5A). The quiescent B-cell cluster (C10) exhibited relatively high expression of *Sell*, *Ccr7*, and *Klf2* (Figure S5A). Because cluster identities were determined by unsupervised clustering of global transcriptional profiles, C10 was annotated as quiescent B cells, reflecting a quiescent transcriptional state that may partially overlap with the naive B-cell compartment, whereas C0 and C4 represented transcriptionally distinct follicular B-cell states rather than discrete developmental subsets. MZ B cells (C2) were identified by *Cd1d1*, *Cd9*, and *Cr2* expression. Activated memory-like B cells (C3) were enriched for immediate-early activation genes, including *Jun*, *Fos*, *Nfkbia*, and *Cd69*, together with genes commonly associated with memory B-cell programs, including *Hhex*, *Tle3*,^29^ *Cd81*, and *Tnfrsf13b* (Figure S5A). PreGC B cells (C5) expressed *Mif*, *Npm1*, and *Eif5a*.^30^ Two GC-associated B-cell populations were identified, corresponding to cycling DZ-like and LZ-like GC B cells. The cycling DZ-like GC B-cell cluster was characterized by expression of proliferative and GC-associated genes, including *Cxcr4*, *Mki67*, *Top2a*, *S1pr2*, and *Bcl6*, whereas the LZ-like GC B-cell cluster preferentially expressed LZ-associated activation genes, including *Cd83* and *Cd86*. *Bcl6* and *Aicda* were enriched within GC the cycling DZ-like cluster. Plasmablast populations included early IgA plasmablasts (C6), characterized by *Jchain* expression, and mature plasmablasts (C12), defined by high *Irf4*, *Xbp1*, *Jchain*, *Slpi*, *Sdc1*, and *Prdm1* expression (Figure S5A). Two atypical B-cell populations were identified: a CD11c^+^ atypical cluster (C9) expressing *Itgax* and *Zeb2*, and a CD11b^+^ atypical cluster (C11) expressing *Itgam*. A B1a population (C14) was resolved based on canonical CD5 expression. An activated memory-like B-cell population (C3) was markedly enriched in HZ mice and expressed the memory-associated transcription factors *Hhex* and *Tle3*^29^^,^^31^ (Figure 2F) together with immediate-early activation genes (*Jun*, *Fos*, *Nfkbia*, and *Cd69*) (Figure S5A). Comparative analysis across genotypes revealed an increased proportion of activated memory-like B cells in HZ mice, along with the reductions in cycling DZ-like GC, early IgA plasmablast populations (Figure 2D). Notably, genotype-separated UMAP visualization showed that the altered distribution of the mature follicular B-cell population was already apparent under homeostatic conditions, whereas the resting follicular B-cell population was relatively preserved (Figure S4G), suggesting that Slc5a6 deficiency perturbs steady-state follicular B-cell homeostasis before antigen challenge. The alum-immunized groups exhibited B-cell subset distributions similar to their corresponding untreated controls and did not show major transcriptional shifts, indicating that alum alone had minimal effects under our experimental conditions (Figure 2D). Biotin supplementation restored GC-associated and plasmablast populations, demonstrating recovery of GC differentiation and downstream B-cell development. Cycling DZ-like GC B cells and early IgA plasmablasts contained the highest proportions of S- and G2/M-phase cells, reflecting their highly proliferative nature. In contrast, the remaining B-cell populations display mixed cell-cycle distributions with a predominance of G1-phase cells. (Figure 2E).

Parallel FlowSOM-based analysis^32^ of the available splenic T cell flow cytometry dataset identified major T cell populations, including pan T cells, CD4 helper T cells, regulatory T cells, naive T cells, and a follicular helper T cell (Tfh) population (Figures S6A and S6B). Comparable representation of the Tfh population was observed among WT, HZ, and HZ_b7 mice, with no major differences in cell abundance between groups (Figure S6C), indicating no major alterations in baseline Tfh cell abundance.

In contrast, expression of terminal plasmablast differentiation genes (*Jchain*, *Xbp1*, *Irf4*, and *Prdm1*) was reduced in HZ samples (Figures 2G and 2H), consistent with impaired plasmablast differentiation. Because the plasmablast population was markedly depleted in HZ mice, these expression differences likely reflect both reduced cell abundance and altered transcriptional activity within the remaining cells. Taken together, these findings suggest that Slc5a6 mutation impairs follicular B-cell maturation and GC progression, with a relative increase in an activated memory-like B-cell population, while no major alterations in splenic T cell subset composition were observed. Restoration of these phenotypes following biotin supplementation further implicates a critical role for biotin in maintaining effective B-cell differentiation and humoral immunity.

### *Slc5a6* mutation creates a developmental blockade at the preGC stage

Single-cell transcriptomics integrated with RNA velocity provides a high-resolution framework to resolve dynamic transitions between GC B cell states, including movement between LZ and DZ compartments that are difficult to infer by flow cytometry alone. To identify differentiation bottlenecks imposed by *Slc5a6* mutation, we performed RNA velocity analysis using CellRank^33^ across experimental conditions. RNA velocity analysis revealed dynamic trajectories toward the early IgA plasmablast cluster (C6) in WT mice following vaccination, indicating active commitment to early plasmablast differentiation (Figure 3A). This directional flow was markedly attenuated in HZ mice but largely restored by biotin supplementation in HZ_b7 mice. Because the mature plasmablast cluster (C12) contained substantially fewer cells than C6, sparse velocity vectors precluded robust trajectory inference for this population. In parallel, WT mice exhibited robust directional transitions from preGC B cells (C5) toward the cycling DZ-like GC B population (C8), consistent with productive GC entry and proliferative expansion (Figure 3B). Notably, the preGC population occupied an intermediate position in the UMAP embedding, spanning the region between the follicular B cell and cycling DZ-like GC B-cell populations. Together with the RNA velocity analysis, this distribution suggests the interpretation that preGC cells represent a transitional state during GC commitment rather than a homogeneous cell population. A comparable transition was observed in HZ_b7 mice, whereas HZ mice exhibited markedly reduced velocity flux toward the cycling DZ-like GC compartment. Although the cycling DZ-like GC B-cell population was substantially reduced in HZ mice, sufficient cells remained for trajectory inference, which revealed attenuated progression toward the cycling DZ-like GC state and increased retention within the preGC compartment. Simulated fate probability mapping further showed these findings by demonstrating increased retention of cells within the preGC state in HZ mice compared with WT and HZ_b7 mice (Figure S7A). Collectively, these results indicate that biotin deficiency impairs both early plasmablast differentiation and GC progression following vaccination, whereas biotin supplementation largely restores these developmental trajectories.

**Figure 3:**
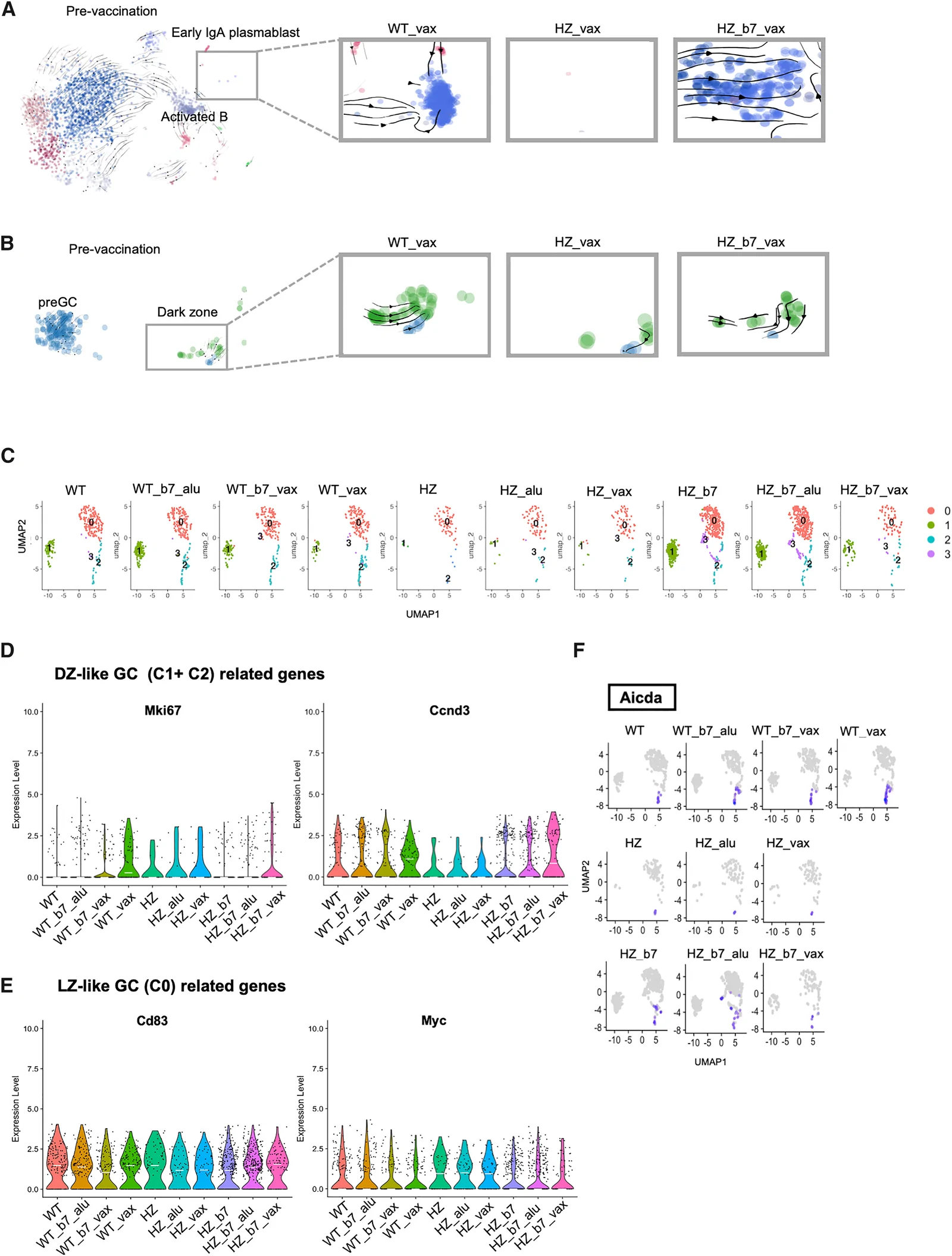
*Slc5a6* mutation disturbs preGC-to-GC transition and interrupts GC zone dynamics WT_vax, HZ_vax, and HZ_b7_vax denote vaccinated WT, HZ, and HZ_b7 mice, respectively. (A) RNA velocity projections illustrating differentiation trajectories toward the early IgA plasmablast population (C6) in WT_vax, HZ_vax, and HZ_b7_vax mice. Arrows indicate directional flow from late follicular/activated B-cell states toward the early IgA plasmablast cluster (C6). (B) RNA velocity projections illustrating transitions from preGC B cells (C5) toward the cycling DZ-like GC B population (C8). (C) UMAP showing GC-related subclusters representing preGC, LZ-like GC, and cycling DZ-like GC states. (D) Violin plots of *Mki67* and *Ccnd3* expression in DZ-associated clusters (clusters 1 and 2) across all conditions. (E) Violin plots of *Cd83* and *Myc* expression in the LZ-associated cluster (cluster 0) across all condition. (F) Feature plot of *Aicda* expression in GC subclusters across all condition.

To refine lineage relationships, we resolved four GC-related subclusters encompassing transitions from preGC through LZ-like and cycling DZ-like GC states (Figure 3C). In line with impaired cycling DZ-like GC development, genes associated with the DZ program, including *Cxcr4*, *Foxo1*,^20^ *Mki67*, and *Ccnd3*^34^ showed markedly reduced overall representation in HZ mice, which was largely restored following biotin supplementation (Figures 3D, S7B, and S7C). In contrast, expression of LZ-associated genes, including *Cd83* and *Myc*,^35^ remained detectable across conditions (Figure 3E), suggesting that acquisition of LZ-associated features is relatively preserved, whereas progression into the proliferative DZ program is selectively impaired. Likewise, *Aicda*-expressing cells were markedly reduced in HZ mice and recovered following biotin supplementation (Figure 3F). Together with the reduced abundance of cycling DZ-like GC B cells, these findings reflect impaired establishment and maintenance of the DZ program under biotin deficiency, providing a mechanistic explanation for reduced somatic hypermutation (SHM), affinity maturation, and downstream plasmablast differentiation.

### Biotin supplementation restores isotype switching, SHM, and BCR repertoire diversity impaired by *Slc5a6* mutation

Upon the observed impairments in GC development and plasmablast differentiation in *Slc5a6* mutant mice, we next evaluated whether these defects extended to downstream humoral immune responses, including immunoglobulin class switching, SHM, and BCR repertoire remodeling. Analysis of IgG:IgM and IgA:IgM ratios showed a trend toward reduced class switching in HZ mice compared with WT and biotin-supplemented HZ_b7 mice (Figure 4A). As expected, our previous study demonstrated that naive B cells from HZ mice exhibit reduced switching to IgG1 under defined *in vitro* stimulation conditions, with a similar trend observed for IgE switching (see Figure 5D in ref;^23^), showing an intrinsic defect in class-switching capacity in *Slc5a6*-deficient B cells. Furthermore, SHM frequency was significantly decreased in HZ mice, aligned with impaired affinity maturation (Figure 4B). To investigate antigen-driven B cell clonal dynamics, we performed BCR repertoire analysis across experimental conditions. At baseline, WT and HZ_b7 mice exhibited diverse and evenly distributed clonal architectures, whereas HZ mice displayed oligoclonal skewing with overrepresentation of a few dominant clones, suggesting reduced repertoire diversity (Figure 4C, upper image). After immunization, WT and HZ_b7 mice demonstrated robust clonal expansion and emergence of large, dominant clones, indicative of effective antigen selection. In contrast, HZ mice failed to undergo this expansion, instead showing a fragmented clonal pattern enriched for small clones (Figure 4C, lower image). Quantitative analysis confirmed this defect in clonal expansion: large clones (≥10 cells) were abundant in WT and HZ_b7 mice, while HZ mice remained skewed toward singletons and small clones (Figure 4D). In vaccinated WT and HZ_b7 mice, the V-allele calls IGKV10-96∗01 and IGKV10-96∗07 were detected within the C6 cluster, paired with IGKJ2∗01 and IGKJ1∗01, respectively, in comparisons with HZ mice (Table 1). Similarly, IGHV1-53∗01 was paired with IGHJ2∗01 in WT mice and IGHJ3∗01 in HZ_b7 mice within the C12 plasmablast cluster (Table 1). These related plasmablast-associated V-J combinations were more prominent in WT and HZ_b7 mice than in HZ mice. In addition, IgA^+^ B cells from HZ mice exhibited significantly reduced SHM frequencies, with a trend toward recovery following biotin supplementation, although mutation frequencies varied among other class-switched isotypes, including higher IGHG2B mutation frequency in HZ mice (Figure S7D). Together with the reduced class switching and clonal diversity observed in HZ mice, these findings support an overall impairment in antibody repertoire maturation despite isotype-specific variation in SHM frequency.

**Figure 4:**
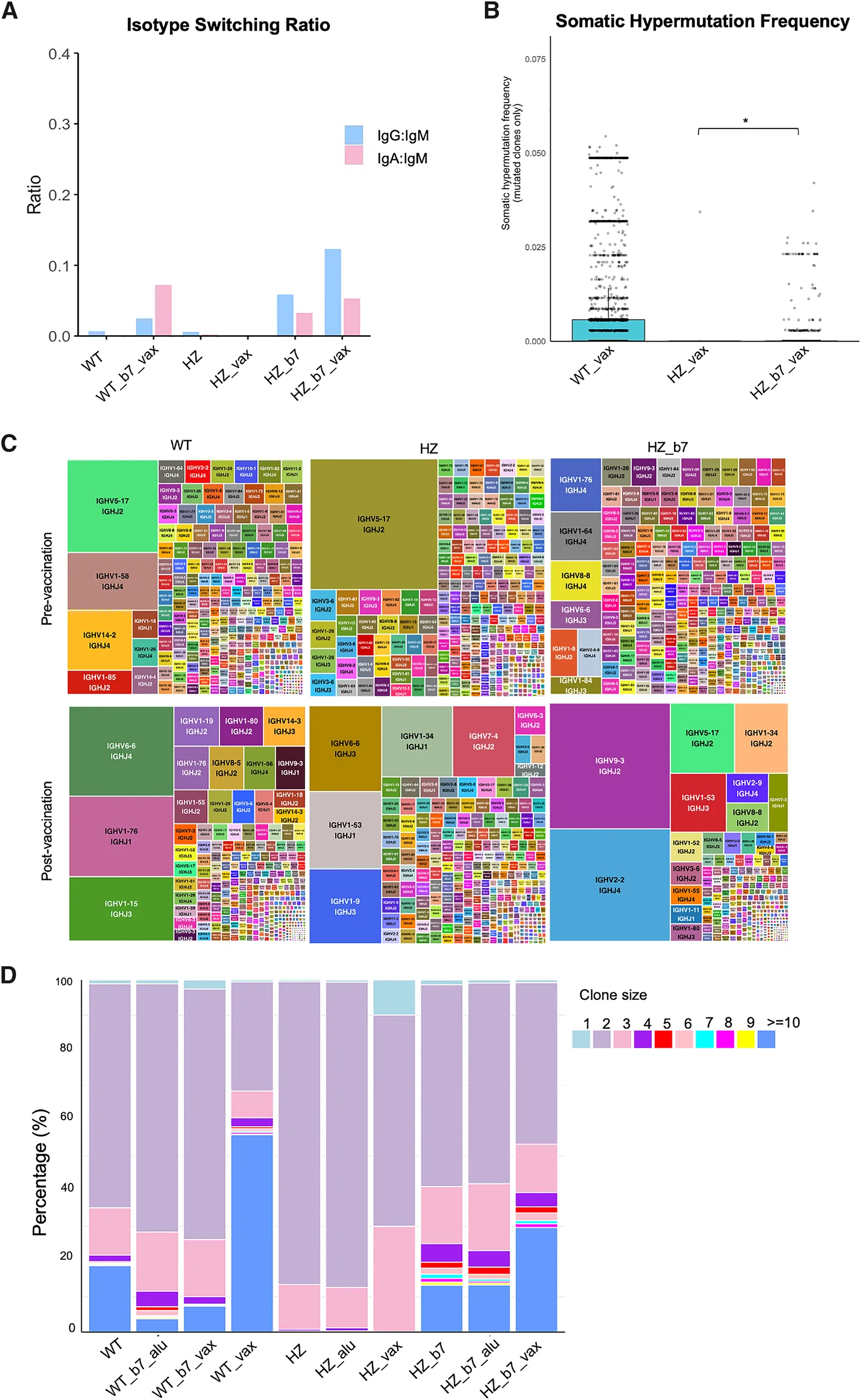
Biotin supplementation restores isotype switching, somatic hypermutation, and clonal expansion defects in *Slc5a6* mutant mice after vaccination (A) Isotype switching ratios of IgG:IgM (blue) and IgA:IgM (pink) before and after vaccination across WT, HZ, and HZ_b7 groups. (B) Somatic hypermutation (SHM) frequencies in B cell populations across experimental conditions. SHM load was calculated among mutation-positive sequences (mu_freq >0) and compared using permutation testing at the sequence level. Statistical significance is indicated by ∗*p* < 0.05. (C) Treemap visualizations of BCR repertoires showing changes in clonal architecture before and after vaccination in WT, HZ, and HZ_b7 mice. Each box represents a unique V–J gene combination. (D) Clone-size distribution analysis comparing the extent of clonal expansion across groups.

**Figure 5:**
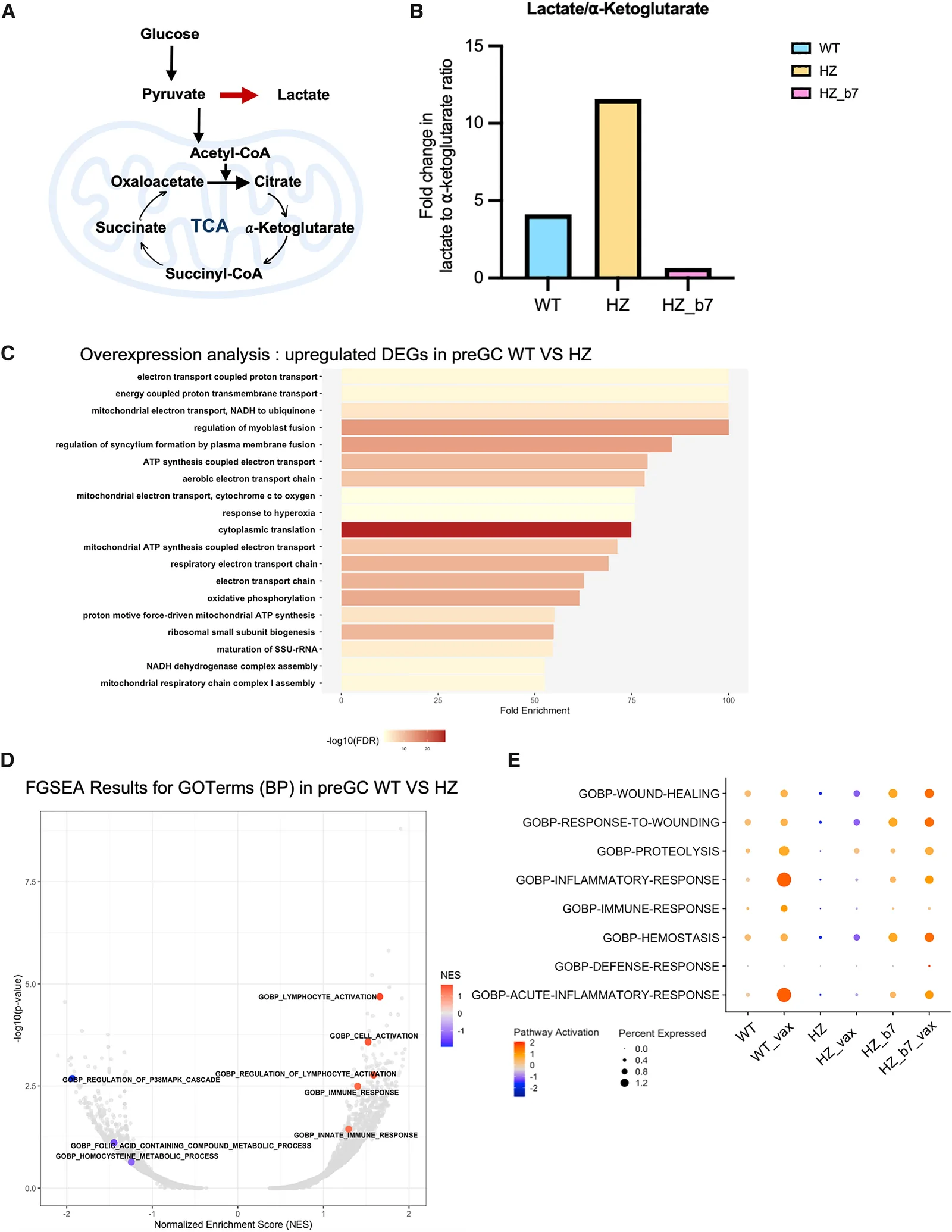
Biotin deficiency reprograms mitochondrial metabolism and alters preGC transcriptional networks in *Slc5a6* mutant B cells (A) Schematic diagram of glycolysis and the TCA cycle branches. The red arrow indicating the conversion of pyruvate to lactate. (B) Fold changes in metabolite ratios (lactate/α-ketoglutarate) in total CD19^+^ CD138^+^B cells from WT, HZ, and HZ_b7 mice. (C) Pathway enrichment from PANTHER over-representation analysis of differentially expressed genes (DEGs) in the preGC (C5) cluster using Gene Ontology biological process (GOBP) terms. FDR-adjusted *p* values were used for statistical significance. (D) Volcano plot showing fast gene set enrichment analysis (FGSEA) results comparing GOBP pathway enrichment between WT and HZ preGC clusters. (E) Dot plot summarizing enriched biological pathways (GOBP) across all experimental conditions.

**Table 1:** V–J gene combinations in B-cell clusters following vaccination

Cluster	Comparison	V allele call	J allele call	log_2_FC
6	WT_vax vs. HZ_vax	IGHV6-6∗01	IGHJ4∗01	7.42
6	WT_vax vs. HZ_vax	IGKV10-96∗01, IGKV10-96∗07	IGKJ2∗01	7.07
6	WT_vax vs. HZ_vax	IGKV8-24∗01	IGKJ5∗01	6.85
6	WT_vax vs. HZ_vax	IGLV1∗01	IGLJ1∗01	6.22
6	WT_vax vs. HZ_vax	IGHV1-56∗01	IGHJ4∗01	5.49
6	HZ_b7_vax vs. HZ_vax	IGKV1-117∗01	IGKJ1∗01	4.85
6	HZ_b7_vax vs. HZ_vax	IGHV1-34∗01	IGHJ2∗01	4.39
6	HZ_b7_vax vs. HZ_vax	IGHV7-3∗01	IGHJ1∗03	3.80
6	HZ_b7_vax vs. HZ_vax	IGKV4-68∗01	IGKJ2∗01	3.58
6	HZ_b7_vax vs. HZ_vax	IGKV10-96∗01, IGKV10-96∗07	IGKJ1∗01	2.58
12	WT_vax vs. HZ_vax	IGHV1-53∗01	IGHJ2∗01	2.00
12	WT_vax vs. HZ_vax	IGHV5-17∗01	IGHJ3∗01	1.58
12	WT_vax vs. HZ_vax	IGKV14-111∗01	IGKJ2∗01	1.58
12	WT_vax vs. HZ_vax	IGLV2∗02	IGLJ2∗01	1.58
12	WT_vax vs. HZ_vax	IGHV1-15∗01	IGHJ3∗01	1.00
12	HZ_b7_vax vs. HZ_vax	IGHV1-53∗01	IGHJ3∗01	1.00
12	HZ_b7_vax vs. HZ_vax	IGHV14-3∗01	IGHJ3∗01	1.00
12	HZ_b7_vax vs. HZ_vax	IGKV3-7∗02	IGKJ1∗01	1.00
12	HZ_b7_vax vs. HZ_vax	IGKV4-59∗01	IGKJ5∗01	1.00
12	HZ_b7_vax vs. HZ_vax	IGKV8-19∗01	IGKJ4∗01	1.00

WT, wild-type mice; HZ, heterozygous *Slc5a6* mutant mice; HZ_b7, biotin-supplemented HZ mice; vax, vaccinated; log_2_FC, log_2_ fold change.

Collectively, these findings suggest that *Slc5a6* mutation impairs GC progression, particularly the cycling DZ-like GC program, and is associated with altered SHM, reduced isotype switching, and restricted BCR repertoire diversification. Biotin supplementation substantially restored these phenotypes, indicating an important role for biotin availability in efficient humoral immune responses.

### Metabolic programs distinguish GC subsets which are disrupted in *Slc5a6* mutant mice

Cluster-wide metabolic profiling using hallmark gene sets revealed distinct metabolic programs across B-cell subsets. The cycling DZ-like GC B-cell cluster (C8) exhibited significantly higher OXPHOS and fatty acid metabolism signatures than the LZ-like GC B-cell cluster (C7), consistent with its proliferative and biosynthetic transcriptional program (Figure S8A). In contrast, hypoxia-associated signatures were preferentially enriched in the CD11b^+^ atypical B-cell subset (C11), suggesting altered metabolic adaptation in this compartment (Figure S8B). These findings indicate that mitochondrial oxidative metabolism is a defining feature of DZ B cells within the GC reaction. Given the preferential reliance of cycling DZ-like GC B-cell on mitochondrial pathways, we next investigated whether *Slc5a6* deficiency perturbs central carbon metabolism. To elucidate metabolic bottlenecks underlying impaired B-cell differentiation in *Slc5a6* mutant mice, we performed LC-MS-based metabolomic profiling on total splenic B cells isolated from WT, HZ, and HZ_b7 mice. Targeted quantification of glycolytic and TCA cycle intermediates revealed a pronounced increase in the lactate-to-α-ketoglutarate ratio in HZ mice (Figure 5B), indicating a shift away from mitochondrial OXPHOS toward glycolytic metabolism. Elevated lactate levels (Figure 5A, red arrow), a hallmark of glycolytic flux, coincided with reduced abundance of key TCA intermediates, including α-ketoglutarate (α-KG) (Figure 5A). These alterations are consistent with impaired anaplerotic replenishment of the TCA cycle.

Because biotin serves as an essential cofactor for multiple carboxylases involved in central carbon metabolism, these metabolite imbalances likely reflect diminished biotin-dependent carboxylation capacity and compromised mitochondrial function. To further define the transcriptional consequences of this metabolic dysregulation, we performed pathway enrichment analyses on the preGC (C5) cluster. Differential expression analysis revealed significant downregulation of mitochondrial gene signatures in HZ mice, including OXPHOS, ATP synthesis-coupled electron transport, and components of the electron transport chain (Figure 5C). Concurrently, one-carbon metabolism and p38 MAPK signaling pathways were significantly enriched in HZ preGC (C5) cells (Figure 5D), in line with activation of compensatory stress-response programs secondary to mitochondrial dysfunction. In contrast to HZ mice, both WT and HZ_b7 groups displayed enrichment of acute inflammatory and immune response pathways following vaccination, which were notably diminished in HZ mice (Figure 5E). Collectively, these findings point to a model in which *Slc5a6* deficiency disrupts mitochondrial metabolic programming, thereby impairing preGC maturation, DZ expansion, and downstream GC responses, whereas biotin supplementation largely restores these metabolic and transcriptional programs.

### Biotin supplementation restores GC-like B cell organization after vaccination

To evaluate splenic architecture and B-cell organization following immunization, we performed high-resolution multiplexed imaging (CODEX) on spleen sections from mutant HZ mice and biotin-supplemented HZ_b7 mice after vaccination. The antibody panel included markers for major B-cell populations (CD19, B220, IgD, CD21/35, CD38, and CD5), T cells (CD3), myeloid cells (CD11b), and GC-associated B cells (GL7), enabling comprehensive visualization of splenic immune architecture. Representative CODEX images of spleens collected following vaccination showed preserved overall splenic organization in both HZ and HZ_b7 mice, including B220^+^ B-cell follicles (green) and CD3^+^ T cell zones (blue) (Figures 6A and S8C for low-magnification overview). In contrast, GL7^+^ activated B-cell regions (magenta) were markedly reduced in HZ mice but readily detectable after biotin supplementation (HZ_b7). Quantitative image analysis demonstrated a higher density of CD19^+^ GL7-high B cells in HZ_b7 mice than in HZ mice (Figure 6B). In addition, GL7 intensity within CD19^+^ GL7-high B cells was significantly increased following biotin supplementation (Figure 6C), indicating enhanced activation of GC-associated B cells. Quantitative analysis further demonstrated a significant increase in the local area occupied by GL7-high GC-associated regions following biotin supplementation (Figure 6D), consistent with enhanced GC organization. Together, these complementary imaging analyses indicate that biotin supplementation promotes the formation and organization of GC-associated B-cell regions following vaccination.

**Figure 6:**
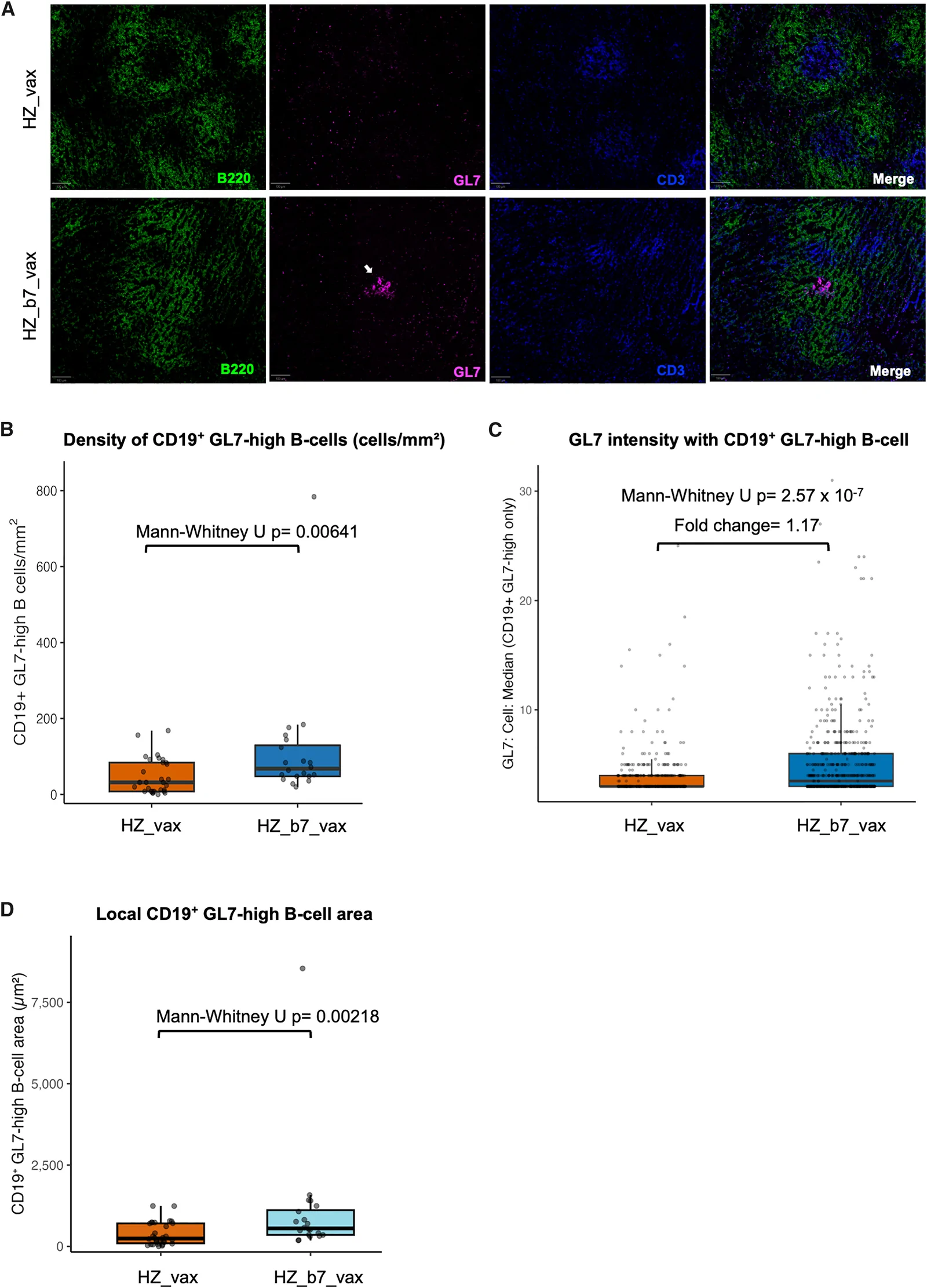
Biotin supplementation restores germinal center-like B cell organization after vaccination Multiplex immunofluorescence/CODEX analyses were performed using spleen sections from HZ_vax mouse and HZ_b7_vax mouse. (A) Representative multiplex immunofluorescence images showing B220^+^ B-cell follicles (green), GL7^+^ activated B cells (magenta; white arrow indicates a GL7-high region), and CD3^+^ T cell zones (blue). Scale bars, 100 μm. (B) Density of CD19^+^ GL7-high B cells within 500 × 500 μm local tissue regions. Each dot represents one local tissue region. (C) GL7 fluorescence intensity measured as the median GL7 intensity of individual CD19^+^ GL7-high B cells. Each dot represents one segmented cell. (D) Local CD19^+^ GL7-high B-cell area, calculated as the summed segmented cellular area of CD19^+^ GL7-high B cells within each 500 × 500 μm local tissue region. Each dot represents one local tissue region. Two-sided Mann-Whitney U tests were applied to region-level measurements (B and D) and cell-level measurements (C).

## Discussion

In this study, we show that mutation of the multivitamin transporter SLC5A6 disrupts mitochondrial metabolism and is associated with impaired B-cell differentiation, defective GC maturation, and reduced antibody-secreting cell development. Single-cell transcriptomics identified a bottleneck at the preGC stage, accompanied by attenuation of GC transcriptional programs and reduced progression toward plasmablast states. Given the normal development of immature B cells in *Slc5a6* mutant mice, these findings implicate a blockade in B-cell maturation along the follicular pathway. They also extend our previous observations in patients with *SLC5A6* mutations and establish the *Slc5a6* M60K mouse as a physiologically relevant *in vivo* model for investigating how biotin-dependent mitochondrial metabolism regulates vaccine-induced humoral immunity.^23^ SLC5A6 transport is compromised, supraphysiologic biotin may restore intracellular biotin availability via passive diffusion or alternative low-affinity uptake pathways at high extracellular concentrations,^36^ explaining the phenotypic rescue observed following biotin supplementation in our model.^23^ Because SLC5A6 transports both biotin and pantothenic acid, we also considered whether altered pantothenic acid availability might contribute to the observed phenotype. In our previous metabolomic analysis, circulating biotin was markedly reduced in *Slc5a6* mutant mice and restored by biotin supplementation, whereas circulating pantothenic acid remained at low concentrations across experimental groups (see Figure S3A in ref;^23^). Although we cannot formally exclude a contribution of altered pantothenic acid metabolism to the mutant phenotype, our data show that biotin supplementation alone is sufficient to rescue the major immunological defects observed in this model. Future studies directly comparing biotin and pantothenic acid supplementation will help define the relative contribution of each SLC5A6 substrate to B-cell biology.

Despite systemic biotin depletion, HZ B cells engaged compensatory stress-adaptive programs, including upregulation of one-carbon metabolism and p38 MAPK signaling in the preGC cluster (Figure 5D). Such responses may reflect attempts to sustain biosynthetic demand under mitochondrial constraint, but they were insufficient to support effective GC entry and cycling. Biotin supplementation restored preGC-to-GC progression, reactivated *Aicda*-associated programs, and enabled plasmablast differentiation. While one-carbon metabolism can support GC formation,^37^ our data highlight a distinct micronutrient-sensitive axis, biotin-dependent carboxylase activity, that operates at an early checkpoint of GC commitment.

Several reports have suggested that early activated and preGC B cells preferentially rely on glucose-mediated pathways.^38^^,^^39^^,^^40^ Our findings refine this model by showing that preGC cells (C5) are concurrently enriched for both glycolysis and OXPHOS hallmark signatures (Figure S8A), providing additional evidence that a metabolically flexible state that engages both cytosolic and mitochondrial programs. Under biotin deficiency, impaired mitochondrial carboxylase activity is likely to constrain oxidative metabolism and anaplerotic support, creating an energetic and biosynthetic bottleneck that glycolysis alone cannot fully compensate. This imbalance may promote stress signaling and reduce the metabolic fitness required for GC entry. In line with the emerging evidence that nutrient availability can influence the balance between GC and EF responses,^21^^,^^41^ our data suggest that micronutrient-dependent mitochondrial function constitutes an important metabolic constraint shaping early GC commitment.

We further identify a connection between mitochondrial integrity and zone-specific GC B-cell function (Figure S8A). GC subclusters exhibited distinct metabolic signatures, with OXPHOS predominating in cycling DZ-like GC B cells. Under biotin deficiency, the dominant defect localized to the cycling DZ-like GC compartment, where anabolic expansion and proliferation were impaired, whereas expression of LZ-associated genes remained detectable (Figure 3E). Because SHM and repertoire diversification require repeated DZ re-entry and proliferation, impaired cycling DZ-like GC development resulting from altered metabolic reprogramming may contribute to the reduced SHM and clonal expansion observed in mutant mice (Figures 4B–4D). Restoration of IgG/IgA switching and SHM following biotin supplementation (Figures 4A and 4B) further highlights a role for biotin-dependent mitochondrial metabolism in sustaining GC function. Interestingly, the marked expansion of the activated memory-like B-cell population in mutant mice suggests that impaired GC progression may divert activated B cells into an alternative transcriptional state characterized by memory-associated features. Together with the RNA velocity and CellRank analyses demonstrating reduced progression toward the cycling DZ-like GC program and increased retention within the preGC state, these findings raise the possibility that biotin deficiency favors an alternative activation trajectory, potentially involving GC-independent or EF pathways. Future lineage-tracing studies will be required to determine the developmental origin and long-term stability of this activated memory-like B-cell population under biotin limitation.

Functionally, HZ mice failed to mount robust NAb responses after SARS-CoV-2 spike protein vaccination, including diminished cross-variant reactivity against Omicron BA.2 (Figure 1E). Consistent with this functional defect, flow cytometric analysis revealed reduced frequencies of total RBD-specific B cells and RBD-specific GL7^+^CD95^+^ GC B cells, together with lower representation of RBD-specific IgG1^+^ and CD19^low/int^CD138^hi^ plasmablast-like populations (Figures S3F–S3I). Biotin supplementation increased these antigen-specific B-cell populations toward WT levels and restored vaccine-induced NAb responses, highlighting the translational relevance of biotin-dependent metabolic regulation for humoral immunity.

Collectively, our study identifies biotin availability as an important regulator of GC output and downstream effector B-cell differentiation. By integrating single-cell transcriptomics, trajectory analysis, BCR repertoire profiling, and spatial imaging, we show that biotin-dependent mitochondrial metabolism facilitates progression through the preGC and cycling DZ-like GC programs associated with effective isotype switching, SHM, and plasmablast differentiation. These findings link systemic micronutrient availability to the metabolic programs that shape vaccine-induced humoral immunity and suggest that metabolic intervention may improve antibody responses under conditions of nutritional insufficiency or metabolic stress.

### Limitations of the study

Although our findings collectively support an important role for *Slc5a6*-dependent vitamin transport in GC B-cell differentiation and humoral immune responses, the present study does not establish whether the impaired GC B-cell response is entirely B-cell intrinsic, as *Slc5a6* deficiency is present systemically and may also affect other cell types that participate in the process of GC B-cell differentiation to antibody producing cells.

## Resource availability

### Lead contact

Further information and requests for resources should be directed to and will be fulfilled by the lead contact, Chi-Chang Shieh (cshieh@mail.ncku.edu.tw).

### Materials availability

This study did not generate new unique reagents. C57BL/6JNarl and C57BL/6JNarl-Slc5a6 M60K mice were available from the National Center for Biomodels (NCB), Tainan, Taiwan. The MVC COVID-19 vaccine was provided by Medigen Vaccine Biologics Corp., Taiwan, under a material transfer agreement.

### Data and code availability

• The single-cell RNA-seq and BCR-seq data generated in this study have been deposited in the NCBI under BioProject : PRJNA1337006.
• This paper does not report original code.
• Any additional information required to reanalyze the data reported in this paper is available from the lead contact upon request.

## Acknowledgments

The authors acknowledge the Core Research Laboratory and the Laboratory Animal Center of the College of Medicine, National Cheng Kung University, for their technical expertise and valuable discussions. We also thank the National Center for Biomodels (NCB), NIAR, Taiwan, for technical support in contract breeding and testing services. We are grateful to Medigen Vaccine Biologics Corp. for providing the MVC COVID-19 vaccine used in this study. This study was supported by Ministry of Science and Technology (10.13039/501100004663MOST 113-2314-B-006-102-MY3) and National Center for Biomodels (NIAR 111-M14), Taiwan, to C.-C.S.

## Author contributions

Conceptualization, C.-H.H. and C.-C.S.; methodology, C.-H.H., W.J.A., Y.-F.H., Y.-C.T., M.-S.L., H.-H.S., D.-Y.H., D.-B.S., S.-T.J., C.-W.L., C.-A.C., and P.-C.C.; investigation, C.-H.H., Y.-F.H., Y.-C.T., and M.-S.L.; visualization, C.-H.H., W.J.A., and Y.-C.T.; funding acquisition, C.-H.H., Y.-F.H., and C.-C.S.; project administration, C.-H.H. and Y.-F.H.; supervision, C.-C.S.; writing – original draft, C.-H.H.; writing – review and editing, C.-H.H., W.J.A., M.W.H., and C.-C.S.

## Declaration of interests

The authors declare no competing interests.

## Declaration of generative AI and AI-assisted technologies in the manuscript preparation process

During the preparation of this work, the authors used ChatGPT (OpenAI) to assist with language editing and refinement of manuscript text. After using this tool, the authors carefully reviewed and edited all content as needed and take full responsibility for the accuracy and integrity of the published article.

## STAR★Methods

### Key resources table

**Table:** 

REAGENT or RESOURCE	SOURCE	IDENTIFIER
**Antibodies**		

Anti-mouse CD19 antibody	BioLegend	Cat# 115541
Anti-mouse B220/CD45R antibody	BD Biosciences	Cat# 565631
Anti-mouse CD21/35 antibody	BioLegend	Cat# 123434
Anti-mouse CD23 antibody	BioLegend	Cat# 101637
Anti-mouse GL7 antibody	BioLegend	Cat# 144613
Anti-mouse CD95/Fas antibody	BioLegend	Cat# 152623
Anti-mouse CD93 antibody	BioLegend	Cat# 136507
Anti-mouse IgM antibody	BioLegend	Cat# 406547
Anti-mouse IgD antibody	BioLegend	Cat# 405749
Anti-mouse IgG1 antibody	BD Biosciences	Cat# 550874
Anti-mouse CD43 antibody	BioLegend	Cat# 143213
Anti-mouse CD138 antibody	BioLegend	Cat# 142545
Anti-mouse TACI antibody	BioLegend	Cat# 133403
Anti-mouse CD44 antibody	BioLegend	Cat# 103031
Anti-mouse CD62L antibody	BioLegend	Cat# 104411
Anti-mouse ICOS/CD278 antibody	BioLegend	Cat# 313558
Anti-mouse CD3 antibody	BioLegend	Cat# 100232
Anti-mouse CD4 antibody	BioLegend	Cat# 100489
Anti-mouse CD8 antibody	BioLegend	Cat# 100739
Anti-mouse CTLA-4/CD152 antibody	BioLegend	Cat# 106317
Anti-mouse CXCR5 antibody	BioLegend	Cat# 145504
PhenoCycler anti-mouse CD38 antibody, AKYP0032-BX019-Alexa Fluor 750	Akoya Biosciences	PN# 4450013
PhenoCycler anti-mouse CD45R/B220 antibody, AKYP0014-BX010-Alexa Fluor 750	Akoya Biosciences	PN# 4450006
PhenoCycler anti-mouse CD5 antibody, AKYP0016-BX017-Atto 550	Akoya Biosciences	PN# 4250007
PhenoCycler anti-mouse IgD antibody, AKYP0030-BX016-Alexa Fluor 750	Akoya Biosciences	PN# 4450012
PhenoCycler anti-mouse CD21/35 antibody, AKYP0037-BX023-Atto 550	Akoya Biosciences	PN# 4250015
PhenoCycler anti-mouse CD19 antibody, AKYP0033-BX020-Atto 550	Akoya Biosciences	PN# 4250014
PhenoCycler anti-mouse CD3 antibody, AKYP0035-BX021-Alexa Fluor 647	Akoya Biosciences	PN# 4550109
PhenoCycler anti-mouse CD11b antibody, AKYP0040-BX025-Alexa Fluor 750	Akoya Biosciences	PN# 4450015
Anti-mouse ICOS/CD278 antibody, clone C398.4A, conjugated in-house to BX001-Atto 550	BioLegend	Cat# 313502; clone C398.4A; BX001-Atto 550 conjugated in-house
Anti-mouse GL7 antibody, clone GL-7, conjugated in-house to BX015-Alexa Fluor 647	Novus Biologicals	Cat# NBP2-00362; clone GL-7; BX015-Alexa Fluor 647 conjugated in-house

**Chemicals, peptides, and recombinant proteins**		

Zombie Fixable Viability Dye B550^TM^	BioLegend	Cat# 423122
Streptavidin-PE	BioLegend	Cat# 405203
Streptavidin-Alexa Fluor 488	BioLegend	Cat# 405235
Medigen SARS-CoV-2 vaccine	Medigen Vaccine Biologics	SP2202V
Alum	InvivoGen	Cat# vac-alu-250
Biotinylated recombinant SARS-CoV-2 spike RBD	BioLegend	Cat# 793904
AffiniPure F(ab')2 goat anti-mouse IgG + IgM (H + L)	Jackson ImmunoResearch	Cat# 115–006–068
Recombinant mouse IL-4	PeproTech	Cat# 214–14
CellTak Cell and Tissue Adhesive	Corning	Cat# 354240
Oligomycin	Sigma-Aldrich	Cat# O4876
FCCP	Sigma-Aldrich	Cat# C2920
Rotenone	Sigma-Aldrich	Cat# R8875
Antimycin A	Sigma-Aldrich	Cat# A8674

**Critical commercial assays**		

CD19 MicroBeads, mouse	Miltenyi Biotec	Cat# 130–121–301
CD138^+^ Plasma Cell Isolation Kit	Miltenyi Biotec	Cat# 130–092–530
Chromium Next GEM Single Cell 5′ Reagent Kit v2 (Dual Index)	10x Genomics	Cat# 1000263
Chromium Single Cell Mouse BCR Amplification Kit	10x Genomics	Cat# 1000255
SARS-CoV-2 Spike Protein Total Antibody ELISA kit	Elabscience	Cat# E-EL-E607

**Deposited data**		

Single-cell RNA-seq data	This paper	NCBI BioProject: PRJNA1337006
BCR-seq repertoire data	This paper	NCBI BioProject: PRJNA1337006

**Experimental models: Organisms/strains**		

Mouse: C57BL/6JNarl	National Center for Biomodels	N/A
Mouse: *Slc5a6* M60K heterozygous knock-in	National Center for Biomodels	N/A

**Software and algorithms**		

Cell Ranger v7.1	10x Genomics	https://www.10xgenomics.com/support/software/cell-ranger
Seurat v5.1.0	Hao et al.^42^	https://satijalab.org/seurat/
DoubletFinder	McGinnis et al.^43^	https://github.com/chris-mcginnis-ucsf/DoubletFinder
Nebulosa v1.15.0	Alquicira-Hernandez et al.^44^	https://github.com/powellgenomicslab/Nebulosa
Scanpy v1.11.5	Wolf et al.^45^	https://scanpy.readthedocs.io/
scVelo v0.3.3	Bergen et al.^46^	https://scvelo.readthedocs.io/
CellRank v2.0.7	Weiler et al.^47^	https://cellrank.org/
escape v2.2.2	Borcherding et al.^48^	https://github.com/BorchLab/escape
immunarch v0.9.1	Popov et al.^49^	https://immunarch.com/
Immcantation/Change-O	Gupta et al.^50^	https://immcantation.org/
FlowJo v10.8.1	BD Biosciences	https://www.flowjo.com/
QuPath v0.5.1	Bankhead et al.^51^	https://qupath.github.io/
Cellpose	Stringer et al.^52^	https://www.cellpose.org/
StarDist	StarDist developers	https://github.com/stardist/stardist
R v4.4.0	R Foundation for Statistical Computing	https://www.r-project.org/
Python v3.11.14	Python Software Foundation	https://www.python.org/

**Other**		

Cytek Northern Lights spectral flow cytometer	Cytek Biosciences	N/A
Chromium Controller	10x Genomics	N/A
NovaSeq X Plus	Illumina	N/A
Seahorse XF extracellular flux analyzer	Agilent	N/A
LCMS-8045	Shimadzu	N/A
PhenoCycler (CODEX) system	Akoya Biosciences	N/A

### Experimental model and study participant details

C57BL/6JNarl mice were obtained from the National Center for Biomodels (NCB) and maintained under specific-pathogen-free conditions. All experimental procedures were approved by the Institutional Animal Care and Use Committee of NCB (IACUC Approval No. NLAC(TN)-111-M-009) and National Cheng Kung University (NCKU; IACUC Approval No. 113129). Due to the embryonic lethality of homozygous Slc5a6 M60K mice, heterozygous mice were used in this study. For biotin supplementation experiments, heterozygous mice receiving biotin-enriched water were derived from mothers that had been provided with 0.5% biotin water throughout their lifetime. After birth, these offspring continued to receive biotin-enriched water. Both male and female mice were used. The study was not designed or powered to evaluate sex-specific effects, and sex was not included as a variable in the statistical analyses. At 6–8 weeks of age, WT, HZ, and biotin-supplemented heterozygous (HZ_b7) mice were assigned to untreated, alum-only, or Medigen SARS-CoV-2 vaccine groups. Serum samples were collected before immunization (day 0). Mice in the immunization groups received a booster injection on day 21 and were euthanized on day 35. Serum and spleens were collected on day 35 for downstream analyses. Upon study completion, animals were humanely euthanized by CO_2_ overexposure following SOP VD1002 and AVMA guidelines (2020), conducted by veterinarian-authorized technical staff. Death was confirmed by the absence of pulse, respiration, corneal reflex, and response to firm toe pinch, together with gray discoloration of the mucous membranes. Cardiac blood was collected thereafter, followed by scheduled tissue harvesting. Animal carcasses were carefully wrapped and collected by qualified licensed waste vendors.

### Method details

#### Generation of *Slc5a6* M60K mice

The *Slc5a6* M60K knock-in mouse was generated as previously described.^23^ Briefly, the human *SLC5A6* mutation (p.M61K) was mapped to the orthologous mouse *Slc5a6* locus (p.M60K). A synthetic single guide RNA (sgRNA: uaccguuggugaacugcuga) targeting exon 1 was co-injected with Cas9 mRNA and a single-stranded oligodeoxynucleotide repair template into C57BL/6J zygotes. Founder mice were screened by PCR using primers (forward: ATCAGCACCGTGAGGATGAG; reverse: TCATAGGCGCTGGTCAGATG) and validated by Sanger sequencing. Successful editing was confirmed by Sanger sequencing (Figure S1A). Heterozygous founders were backcrossed for at least three generations to minimize off-target effects. Homozygous mutants were not viable due to embryonic lethality.

#### Genotyping of *Slc5a6* M60K mice

Genotyping was performed by PCR amplification of the targeted *Slc5a6* locus using primers (forward: ATCAGCACCGTGAGGATGAG; reverse: TCATAGGCGCTGGTCAGATG), generating a 404 bp amplicon. PCR products were subjected to restriction enzyme digestion (BccI; restriction site: 5′-CCATC(N)4↓-3′), which distinguishes wild-type and mutant alleles based on the presence or disruption of the restriction site. Following digestion, WT alleles produced three fragments (186, 133, and 85 bp), whereas the mutant allele lacking the restriction site is predicted to generate two fragments (271 and 133 bp). HZ mice displayed a mixed banding pattern consistent with the presence of both alleles (Figure S1B).

#### Flow cytometric identification of B and T cell subsets

Murine spleen and bone marrow cells were isolated as previously described.^53^ Single-cell suspensions were prepared by mechanical dissociation, followed by filtration through 40 μm cell strainers. A total of 5 × 10^5^ cells per sample were stained with fluorescently conjugated antibodies in FACS buffer (PBS supplemented with 2% FBS) for 30 min on ice. After staining, cells were washed twice and resuspended in FACS buffer for acquisition using a Cytek Northern Lights flow cytometer (Cytek Biosciences). Viability was assessed using Zombie Fixable Viability Dye (B550, BioLegend, Cat. 423122). Data were analyzed using FlowJo v10.8.1 (BD Biosciences). Lymphocytes were identified based on forward and side scatter, followed by singlet and live-cell gating (80–90% viability). B-cell subsets were defined using the following gating strategy (Detailed flow cytometry gating strategies for all analyzed populations are provided in Figures S2A and S2B). In the spleen, follicular B cells were identified as CD19^+^ B220^+^ CD21^+^ CD23^+^, and marginal zone B cells as CD19^+^ B220^+^ CD21^+^ CD23^−^. Germinal center B cells were defined as CD19^+^ B220^+^ GL7^+^ CD95^+^. Transitional B-cell subsets were characterized as follows: T1 (CD19^+^ B220^+^ CD93^+^ IgM^+^ CD23^−^), T2 (CD19^+^ B220^+^ CD93^+^ IgM^+^ CD23^+^), and T3 (CD19^+^ B220^+^ CD93^+^ IgM^−^ CD23^+^). B1 cells were identified as CD19^+^ CD43^+^. In the bone marrow, plasma cells were defined using a multiparameter gating strategy incorporating CD138, TACI, CD19, and B220, following established protocols.^24^

For T cell phenotyping, splenic T cells were identified as CD19^-^B220^−^CD3^+^ lymphocytes. FlowSOM clustering was performed on gated T cells, and cell populations were annotated based on canonical marker expression. Pan T cells were defined as CD19^-^B220^−^CD3^+^ cells, CD4 helper T cells as CD3^+^CD4^+^ cells, Tfh cells as CD3^+^CD4^+^CXCR5^+^ICOS (CD278)^+^ cells, regulatory T cells as CD3^+^CD4^+^CTLA-4 (CD152)^+^ cells, and naive T cells as CD3^+^CD62L^+^CD44^−^ cells.

The antibody panel included: CD19 (BV650, BioLegend, Cat. 115541), B220 (BV480, BD Biosciences, Cat. 565631), CD21/35 (APC-Fire 750, BioLegend, Cat. 123434), CD23 (BV605, BioLegend, Cat. 101637), CD138 (PE-Fire810, BioLegend, Cat. 142545), GL7 (Pacific Blue, BioLegend, Cat. 144613), CD95 (APC-Fire810, BioLegend, Cat. 152623), CD93 (FITC, BioLegend, Cat. 136507), IgM (Spark Violet 423, BioLegend, Cat. 406547), CD43 (Alexa Fluor 700, BioLegend, Cat. 143213). Plasma-cell markers included TACI (PE, BioLegend, Cat. 133403). T cell antibody panel consisted of CD44 (PerCP-Cy5.5, BioLegend, Cat. 103031), CD62L (APC, BioLegend, Cat. 104411), ICOS/CD278 (BV750, BioLegend, Cat. 313558), CD3 (BV785, BioLegend, Cat. 100232), CD4 (Spark Blue 574, BioLegend, Cat. 100489), CD8 (BV570, BioLegend, Cat. 100739), CXCR5 (PE, BioLegend, Cat. 145504) and CTLA-4/CD152 (PE-Dazzle 594, BioLegend, Cat. 106317).

#### Single cell library preparation and sequencing

Splenic B cells were collected from WT, HZ, and HZ_b7 mice, with 3–4 individual mice pooled per experimental condition to generate one biological sample for single-cell RNA sequencing. Each pooled sample was processed independently for B-cell enrichment and library preparation. Total splenic B-lineage cells were enriched using a sequential magnetic separation strategy. CD19^+^ B cells were first isolated from total splenocytes using Miltenyi CD19 MicroBeads (Cat. 130–121–301) according to the manufacturer’s instructions. The CD19^−^ flow-through fraction was subsequently processed using a non-plasmablast/plasma cell depletion followed by CD138^+^ positive selection (Miltenyi, Cat. 130–092–530) to enrich for plasmablasts and plasma cells. The CD19^+^ and CD138^+^ fractions were then combined and immediately processed for single-cell RNA sequencing. Total fraction of live cells was counted, and cell suspensions having at least 80% viability were processed for scRNA-seq using the Chromium Next GEM Single Cell 5′ Reagent Kits v2 (Dual Index) (10x Genomics, PN-1000263). For BCR repertoire analysis, the Chromium Single Cell Mouse BCR Amplification Kit (10x Genomics, PN-1000255) was used. Sequencing libraries were prepared according to the manufacturer’s guidelines and sequenced on Illumina NovaSeq X Plus (Illumina, California, USA).

#### Single cell bioinformatic analyses

Raw sequencing reads were processed using 10x Genomics Cell Ranger v7.1 to generate gene expression and BCR feature matrices. Per-sample gene expression matrices were parsed in R v4.4.0 using Seurat v5.1.0.^42^ Doublets were identified and removed using DoubletFinder with parameters optimized by in-house scripts.^43^ Standard quality-control filtering was applied to exclude low-quality cells based on gene complexity and mitochondrial content, retaining cells with 200–4000 detected genes, a log10 genes-per-UMI ratio ≥0.8, and mitochondrial gene expression ≤10%, consistent with established best practices.^54^ In practice, the retained cells in our dataset exhibited substantially lower mitochondrial content (0.00–4.99%), indicating high overall data quality. Genes detected in fewer than 10 cells were excluded from downstream analyses. To ensure high-confidence B-lineage assignment, contaminating non–B-lineage cells were removed prior to final clustering. Cells lacking canonical B-cell markers (*Cd19*, *Cd79a*, *Cd79b*, *Pax5*) or expressing lineage-defining genes associated with T cells (e.g., *Cd3e*, *Cd4*, *Cd8a*), myeloid cells (e.g., *Lyz2*, *Itgam*), dendritic cells (e.g., *Itgax*), or stromal compartments were excluded. Importantly, cells expressing plasmablast-associated genes (*Irf4*, *Xbp1*, *Prdm1*, *Jchain*, *Sdc1*) were retained irrespective of surface marker downregulation to preserve terminally differentiated B-lineage states. Following lineage filtering, the dataset was re-normalized and re-clustered for downstream analyses. Gene expression values were log-normalized using the NormalizeData function in Seurat,^55^ and datasets were integrated using the CCA-based integration framework implemented in Seurat v5.1.0.^56^ Cell distances were computed using k-nearest neighbors to construct a shared nearest-neighbor graph, and clusters were identified at a resolution of 0.25 based on global transcriptomic signatures characteristic of B-lineage cells. Cluster proportions were calculated using in-house scripts. Quality-control metrics and canonical marker validation, including mitochondrial read fractions, gene and UMI distributions, and B-lineage and germinal center marker expression, are shown in Figure S3.

#### Germinal center B cell analysis

For lineage identity and differentiation states across single cells, the scaled expression of marker genes was computed and visualized using Seurat’s DotPlot function. Expression of low-signal genes were computed and visualized using a weighted kernel density method in Nebulosa v1.15.0.^44^ GC B cells (preGC, dark zone, and light zone clusters) were extracted and re-clustered using a resolution of 0.2. For lineage trajectory analysis, the Seurat object was converted to AnnData format^57^ and parsed in Python v3.11.14 using Scanpy v1.11.5.^45^ Per-cell transition probabilities were computed by correlating velocity vectors obtained from scvelo v0.3.3^46^ with gene expression differences for each cell and its nearest neighbors using the VelocityKernel in CellRank v2.0.7.^47^ The resulting transition matrix was combined with a similarity-based transition matrix computed using the built-in Connectivity Kernel. Random walks were simulated to visualize transitions starting with preGC cells toward dark zone and light zone B cells.

#### Pathway and functional enrichment analysis

Single-sample Gene Set Enrichment Analysis (ssGSEA) in escape v2.2.2^48^ was performed using the mouse M5 biological process (BP) gene set library^58^^,^^59^ to assess functional differences between clusters. Differentially expressed genes with positive log2 fold change values and adjusted *p*-values less than 0.05 were used for overrepresentation analysis of biological process gene ontology (GOBP) gene sets in PANTHER (geneontology.org), using Fisher’s exact test and computing false discovery rates (FDR) to adjust *p*-values during multiple testing.

#### Gene expression and B cell repertoire analysis

BCR feature matrices were generated from CellRanger v7.1 and analyzed in R using Immunarch v0.9.1^49^ and Immcantation^50^ tools. GEX cell barcodes were matched with VDJ cell barcodes and unmatched barcodes were excluded from the analysis. Cluster annotation of GEX barcodes were transferred to matching barcodes in the BCR data. Productive clones were identified and grouped based on sample ID and VJ gene usage.

#### Pseudovirus neutralization assay

Mouse serum was collected on day 35 post-immunization. Serum samples were 5-fold serially diluted starting at 1:10 and incubated with pseudovirus in duplicate in a 96-well white-bottom plate for 1 h at 37 °C to allow neutralization reactions between virus particles and neutralizing antibodies. After incubation, 3 × 10^4^ 293T-ACE2 cells were seeded into each well and cultured for 48 h at 37 °C in a humidified incubator with 5% CO_2_. The pseudovirus encodes a luciferase reporter, which allows detection of infection levels via luminescence. Luciferase activity, measured in relative light units (RLUs), was quantified using the Neolite Luminescence Assay Kit according to the manufacturer’s instructions. The percentage of neutralization was calculated using the formula below, where background refers to wells containing only media:

$$\%\text{neutralization}=100-\left(\frac{mean\left[sample\right]RLUs-mean\left[background\right]RLUs}{mean\left[virus\;only\right]RLUs-mean\left[background\right]RLUs}\times 100\right)$$

#### SARS-CoV-2 spike-specific total antibody ELISA

Serum samples were collected on day 35 after immunization. Total antibodies against the SARS-CoV-2 spike protein were detected using the Mouse SARS-CoV-2 Spike Protein Total Antibody ELISA Kit (Elabscience, Cat. E-EL-E607) according to the manufacturer’s instructions. Briefly, serum samples were added to spike protein-coated ELISA plates and incubated as instructed by the manufacturer. Following washing, HRP-conjugated detection reagent was added, followed by TMB substrate for color development. The reaction was terminated with stop solution, and absorbance was measured at 450 nm using a microplate reader. Positive and negative controls supplied with the kit were included in each assay for quality control. The assay cutoff value was calculated as 2.1 × the mean absorbance of the negative control, according to the manufacturer’s protocol.

#### Flow cytometric analysis of RBD-specific B cells

Frozen splenocytes were rapidly thawed at 37°C, diluted in pre-warmed complete RPMI medium, and centrifuged to remove residual cryopreservation medium. Cells were resuspended in complete RPMI and allowed to recover for 30 min at 37°C with 5% CO_2_ before staining. After washing with PBS, cell viability was assessed using Zombie Fixable Viability Dye B550 (BioLegend, Cat. 423122) for 15 min on ice, followed by Fc receptor blockade for 5 min on ice. SARS-CoV-2 RBD-specific B cells were detected using two independently labeled RBD probes generated from biotinylated recombinant SARS-CoV-2 spike receptor-binding domain (RBD; BioLegend, Cat. 793904) complexed separately with PE-conjugated streptavidin (BioLegend, Cat. 405203) or Alexa Fluor 488-conjugated streptavidin (BioLegend, Cat. 405235). RBD and streptavidin were combined at an approximately 4:1 M ratio to generate RBD-PE and RBD-AF488 probes. Based on preliminary probe titration experiments, the low-concentration condition was used for subsequent analyses, corresponding to 10 ng RBD equivalent of each probe per 1 × 10^6^ splenocytes in a 100-μL staining volume, with probe amounts scaled proportionally when larger cell numbers were used. Following Fc blockade, cells were incubated with both RBD probes for 60 min at 4°C protected from light, washed, and subsequently stained for 30 min at 4°C with antibodies against CD19 (BV650, BioLegend, Cat. 115541), B220 (BV480, BD Biosciences, Cat. 565631), GL7 (Pacific Blue, BioLegend, Cat. 144613), CD95 (APC-Fire 810, BioLegend, Cat. 152623), IgD (Spark NIR 685, BioLegend, Cat. 405749), IgM (Spark Violet 423, BioLegend, Cat. 406547), IgG1 (APC, BD Biosciences, Cat. 550874), and CD138 (PE-Fire 810, BioLegend, Cat. 142545). After washing, cells were resuspended in staining buffer, passed through a 40-μm cell strainer, and acquired on a Cytek Aurora spectral flow cytometer (Cytek Biosciences). For analysis, lymphocytes were sequentially gated for singlets and viable cells. B cells were identified as CD19^+^B220^+^ cells, and germinal center B cells were defined as GL7^+^CD95^+^ cells within the CD19^+^B220^+^ B-cell population. RBD-specific cells were identified as dual-positive RBD-AF488^+^RBD-PE^+^ events using a common RBD-DP gate applied consistently across all samples. Total RBD-specific B cells were quantified within CD19^+^B220^+^ B cells, RBD-specific GC B cells within GL7^+^CD95^+^ GC B cells, and RBD-specific IgG1^+^ B cells within the IgG1^+^ CD19^+^B220^+^ B-cell population. For analysis of the CD138-high population, CD19^low/int^ CD138^hi^ cells were identified within viable singlet lymphocytes based on relative CD19 and CD138 expression using the same gating boundaries across samples, and RBD-DP cells were quantified within this population. Frequencies of RBD-DP cells were expressed as events per 1,000 cells of the indicated parent population (‰).

#### Lactate and α-ketoglutarate quantification by LC-MS/MS

Lactate and α-ketoglutarate (α-KG) levels were measured in total splenic B cells using LC-MS/MS (Shimadzu LCMS-8045). Cell pellets were dried under nitrogen gas and reconstituted in 0.3–1 mL of 50% methanol. After vortex and centrifugation (3,000 rpm, 15 min), supernatants were filtered (0.22 μm) for analysis. Lactate (Standard powder (L-(+)-Lithium lactate, Sigma, Lot #S8411784413) was detected using electrospray ionization (negative mode), with ion transitions at m/z 89 → 43 and 89 → 45. Isocratic elution was performed using 10 mM ammonium acetate and acetonitrile (40:60, v/v) at 0.6 mL/min. A 10 μL injection was used, and quantification was based on a standard curve (R^2^ = 0.9864), normalized to 10^6^ cells. α-Ketoglutarate (Standard powder α-Ketoglutaric acid sodium salt, Sigma, Lot #SLCD6876) was analyzed using a ZIC-HILIC column with mobile phases containing ammonium formate. Isocratic elution (MPA: MPB = 5:95) was carried out at 0.4 mL/min, 10 μL injection, and detection in negative mode (m/z 145 → 101 and 145 → 83). The standard curve showed excellent linearity (R^2^ = 0.9996). Final concentrations were normalized per 10^6^ cells.

#### Multiplexed imaging and spatial analysis

Multiplexed immunofluorescence was performed using the PhenoCycler (CODEX, Akoya Biosciences) system on frozen spleen and lymph node sections from mice. Tissues were cryosectioned at 3–5 μm thickness and fixed in chilled acetone prior to staining. A four-cycle antibody panel was applied, with each cycle incorporating four fluorophores (Alexa Fluor 488, Atto 550, Alexa Fluor 647, and Alexa Fluor 750). The antibody panel included the following: Anti-Mu CD38 (AKYP0032)-BX019-Alexa Fluor 750 (PN 4450013), Anti-Mu CD45R/B220 (AKYP0014)-BX010-Alexa Fluor 750 (PN 4450006), Anti-Mu CD5 (AKYP0016)-BX017-Atto 550 (PN 4250007), Anti-Mu IgD (AKYP0030)-BX016-Alexa Fluor 750 (PN 4450012), Anti-Mu CD21/35 (AKYP0037)-BX023-Atto 550 (PN 4250015), Anti-Mu CD19 (AKYP0033)-BX020-Atto 550 (PN 4250014), Anti-Mu CD3 (AKYP0035)-BX021-Alexa Fluor 647 (PN 4550109), Anti-Mu CD11b (AKYP0040)-BX025-Alexa Fluor 750 (PN 4450015), anti-mouse ICOS/CD278 (clone C398.4A, BioLegend, Cat. 313502), conjugated in-house to BX001-Atto 550, and anti-mouse GL7 (clone GL-7, Novus Biologicals, Cat. NBP2-00362), conjugated in-house to BX015-Alexa Fluor 647 for PhenoCycler analysis. After each imaging round, fluorophores were inactivated following Akoya Biosciences standard protocols to permit sequential imaging of additional markers on the same tissue section. Imaging was performed using a 20× objective on the Akoya PhenoCycler system, and image registration, stitching, and background subtraction were carried out using the CODEX Processor software. Single-cell segmentation and spatial marker quantification were performed in QuPath v0.5.1^51^ using the StarDist (https://arxiv.org/abs/1806.03535) and Cellpose^52^ plugins. Resulting annotations and measurements were imported into R for downstream spatial analysis using the cdQuPath R package (https://github.com/jianhong/cdQuPath). CD19^+^ GL7-high B cells were quantified from segmented single-cell CODEX data. CD19 positivity was defined using a fixed threshold of CD19 cell median intensity ≥1. GL7-high cells were defined using the 95th percentile of GL7 cell median intensity among pooled CD19^+^ cells. To generate multiple spatial measurements per specimen, each tissue section was divided into non-overlapping 500 × 500 μm local tissue regions. Regions containing fewer than 20 segmented cells were excluded. For each local region, CD19^+^ GL7-high B-cell density was calculated as the number of CD19^+^ GL7-high cells per mm^2^. Local CD19^+^ GL7-high B-cell area was calculated as the summed segmented cellular area of CD19^+^ GL7-high B cells within each region. GL7 fluorescence intensity was quantified using the median GL7 intensity of individual segmented CD19^+^ GL7-high B cells. No IQR-based filtering was applied. Representative local regions were selected based on their proximity to the median CD19^+^ GL7-high B-cell density within each specimen.

#### Seahorse

Total splenic B cells were stimulated for 18 h with AffiniPure F(ab’)_2_ goat anti-mouse IgG + IgM (H + L) (Jackson ImmunoResearch, Cat. 115–006–068, 10 μg/mL) and IL-4 (PeproTech, Cat. 214–14, 10 ng/mL) prior to metabolic analysis to model early activation. A total of 5 × 10^5^ cells per well were plated onto CellTak-coated XF24 plates (CellTak, Cat. 354240, CORNING, Massachusetts, USA; plates from Agilent, Santa Clara, California, USA). Oxygen consumption rate (OCR) was measured using a Seahorse XF extracellular flux analyzer. A mitochondrial stress test was performed with sequential injections of oligomycin (Sigma-Aldrich, Cat. O4876, 2 μM), FCCP (Sigma-Aldrich, Cat. C2920, 2.5 μM), and rotenone/antimycin A (Sigma-Aldrich, Cat. R8875; Sigma-Aldrich, Cat. A8674, 1 μM). Cell viability and plating consistency were confirmed before each run. Biological replication consisted of three independently analyzed mice per group. Replicate Seahorse wells were used as technical replicates, with five replicate wells included for the quantification of respiratory parameters. Individual mice represented the biological experimental units, whereas replicate wells represented technical measurements.

### Quantification and statistical analysis

Statistical analyses were performed using GraphPad Prism (GraphPad Software) and the analysis software described above. Depending on the experimental design, comparisons were performed using unpaired two-tailed t tests, two-way ANOVA with multiple-comparisons correction, Mann–Whitney U tests, or Kruskal–Wallis tests followed by Dunn’s multiple-comparisons test, as specified in the corresponding figure legends. Fisher’s exact test with false-discovery-rate correction was used for pathway overrepresentation analysis. Exact *n* values, definitions of biological and technical replicates or observational units, and measures of center and dispersion are provided in the corresponding figure legends. Unless otherwise indicated, *p* < 0.05 was considered statistically significant. *p* < 0.05; *p* < 0.01; *p* < 0.001; *p* < 0.0001; ns, not significant.

## References

[1] Hu T., Liu C.H., Lei M., Zeng Q., Li L., Tang H., Zhang N. (2024). Metabolic regulation of the immune system in health and diseases: mechanisms and interventions. Signal Transduct. Target. Ther..

[2] Hu C., Xuan Y., Zhang X., Liu Y., Yang S., Yang K. (2022). Immune cell metabolism and metabolic reprogramming. Mol. Biol. Rep..

[3] Lunt S.Y., Vander Heiden M.G. (2011). Aerobic glycolysis: meeting the metabolic requirements of cell proliferation. Annu. Rev. Cell Dev. Biol..

[4] Vander Heiden M.G., Cantley L.C., Thompson C.B. (2009). Understanding the Warburg effect: the metabolic requirements of cell proliferation. Science.

[5] Ma S., Ming Y., Wu J., Cui G. (2024). Cellular metabolism regulates the differentiation and function of T-cell subsets. Cell. Mol. Immunol..

[6] Xu K., Yin N., Peng M., Stamatiades E.G., Shyu A., Li P., Zhang X., Do M.H., Wang Z., Capistrano K.J. (2021). Glycolysis fuels phosphoinositide 3-kinase signaling to bolster T cell immunity. Science.

[7] Xu K., Yin N., Peng M., Stamatiades E.G., Chhangawala S., Shyu A., Li P., Zhang X., Do M.H., Capistrano K.J. (2021). Glycolytic ATP fuels phosphoinositide 3-kinase signaling to support effector T helper 17 cell responses. Immunity.

[8] Bevilacqua A., Franco F., Lu Y.T., Rahiman N., Kao K.C., Chuang Y.M., Zhu Y., Held W., Xie X., Gunsalus K.C. (2024). PPARbeta/delta-orchestrated metabolic reprogramming supports the formation and maintenance of memory CD8(+) T cells. Sci. Immunol..

[9] Wculek S.K., Dunphy G., Heras-Murillo I., Mastrangelo A., Sancho D. (2022). Metabolism of tissue macrophages in homeostasis and pathology. Cell. Mol. Immunol..

[10] Van den Bossche J., O'Neill L.A., Menon D. (2017). Macrophage Immunometabolism: Where Are We (Going)?. Trends Immunol..

[11] Yan J., Horng T. (2020). Lipid Metabolism in Regulation of Macrophage Functions. Trends Cell Biol..

[12] Johnstone J.C., Yazicioglu Y.F., Clarke A.J. (2024). Fuelling B cells: dynamic regulation of B cell metabolism. Curr. Opin. Immunol..

[13] Fu Y., Wang L., Yu B., Xu D., Chu Y. (2022). Immunometabolism shapes B cell fate and functions. Immunology.

[14] Doughty C.A., Bleiman B.F., Wagner D.J., Dufort F.J., Mataraza J.M., Roberts M.F., Chiles T.C. (2006). Antigen receptor-mediated changes in glucose metabolism in B lymphocytes: role of phosphatidylinositol 3-kinase signaling in the glycolytic control of growth. Blood.

[15] Chen D., Wang Y., Manakkat Vijay G.K., Fu S., Nash C.W., Xu D., He D., Salomonis N., Singh H., Xu H. (2021). Coupled analysis of transcriptome and BCR mutations reveals role of OXPHOS in affinity maturation. Nat. Immunol..

[16] Weisel F.J., Mullett S.J., Elsner R.A., Menk A.V., Trivedi N., Luo W., Wikenheiser D., Hawse W.F., Chikina M., Smita S. (2020). Germinal center B cells selectively oxidize fatty acids for energy while conducting minimal glycolysis. Nat. Immunol..

[17] Sharma R., Smolkin R.M., Chowdhury P., Fernandez K.C., Kim Y., Cols M., Alread W., Yen W.F., Hu W., Wang Z.M. (2023). Distinct metabolic requirements regulate B cell activation and germinal center responses. Nat. Immunol..

[18] Gracie C.J., Mitchell R., Johnstone J.C., Clarke A.J. (2025). The unusual metabolism of germinal center B cells. Trends Immunol..

[19] Young C., Brink R. (2021). The unique biology of germinal center B cells. Immunity.

[20] Victora G.D., Nussenzweig M.C. (2022). Germinal Centers. Annu. Rev. Immunol..

[21] Elsner R.A., Shlomchik M.J. (2020). Germinal Center and Extrafollicular B Cell Responses in Vaccination, Immunity, and Autoimmunity. Immunity.

[22] Ochoa-Ruiz E., Díaz-Ruiz R., Hernández-Vázquez A.d.J., Ibarra-González I., Ortiz-Plata A., Rembao D., Ortega-Cuéllar D., Viollet B., Uribe-Carvajal S., Corella J.A., Velázquez-Arellano A. (2015). Biotin deprivation impairs mitochondrial structure and function and has implications for inherited metabolic disorders. Mol. Genet. Metab..

[23] Hsieh C.H., Lee J., Sung H.H., Huang Y.F., Ding Y.S., Li C.Y., Yen C.L., Hsu C.K., Yu C.K., Hsieh H.Y. (2023). Novel SLC5A6 mutations lead to B lymphocyte maturation defects with metabolic abnormality rescuable by biotin replenishment. Clin. Immunol..

[24] Pracht K., Meinzinger J., Daum P., Schulz S.R., Reimer D., Hauke M., Roth E., Mielenz D., Berek C., Côrte-Real J. (2017). A new staining protocol for detection of murine antibody-secreting plasma cell subsets by flow cytometry. Eur. J. Immunol..

[25] Manu A.A., Owusu I.A., Oyawoye F.O., Languon S., Barikisu I.A., Tawiah-Eshun S., Quaye O., Donkor K.J., Paemka L., Amegatcher G.A. (2024). Development and utility of a SARS-CoV-2 pseudovirus assay for compound screening and antibody neutralization assays. Heliyon.

[26] Morgan D., Tergaonkar V. (2022). Unraveling B cell trajectories at single cell resolution. Trends Immunol..

[27] Kennedy D.E., Okoreeh M.K., Maienschein-Cline M., Ai J., Veselits M., McLean K.C., Dhungana Y., Wang H., Peng J., Chi H. (2020). Novel specialized cell state and spatial compartments within the germinal center. Nat. Immunol..

[28] Lee R.D., Munro S.A., Knutson T.P., LaRue R.S., Heltemes-Harris L.M., Farrar M.A. (2021). Single-cell analysis identifies dynamic gene expression networks that govern B cell development and transformation. Nat. Commun..

[29] Laidlaw B.J., Duan L., Xu Y., Vazquez S.E., Cyster J.G. (2020). The transcription factor Hhex cooperates with the corepressor Tle3 to promote memory B cell development. Nat. Immunol..

[30] Hillion S., Miranda A., Le Dantec C., Boudigou M., Le Pottier L., Cornec D., Torres R.M., Pelanda R. (2024). Maf expression in B cells restricts reactive plasmablast and germinal center B cell expansion. Nat. Commun..

[31] Good-Jacobson K.L., Groom J.R. (2020). Hhex drives B cells down memory lane. Nat. Immunol..

[32] Quintelier K., Couckuyt A., Emmaneel A., Aerts J., Saeys Y., Van Gassen S. (2021). Analyzing high-dimensional cytometry data using FlowSOM. Nat. Protoc..

[33] Lange M., Bergen V., Klein M., Setty M., Reuter B., Bakhti M., Lickert H., Ansari M., Schniering J., Schiller H.B. (2022). CellRank for directed single-cell fate mapping. Nat. Methods.

[34] Ramezani-Rad P., Chen C., Zhu Z., Rickert R.C. (2020). Cyclin D3 Governs Clonal Expansion of Dark Zone Germinal Center B Cells. Cell Rep..

[35] Nakagawa R., Toboso-Navasa A., Schips M., Young G., Bhaw-Rosun L., Llorian-Sopena M., Chakravarty P., Sesay A.K., Kassiotis G., Meyer-Hermann M., Calado D.P. (2021). Permissive selection followed by affinity-based proliferation of GC light zone B cells dictates cell fate and ensures clonal breadth. Proc. Natl. Acad. Sci. USA.

[36] Canda E., Kalkan Uçar S., Çoker M. (2020). Biotinidase Deficiency: Prevalence, Impact And Management Strategies. Pediatric Health Med. Ther..

[37] Wu J., Zhou J., Li G., Sun X., Xiang C., Chen H., Jiang P. (2025). Metabolic determinants of germinal center B cell formation and responses. Nat. Chem. Biol..

[38] Choi S.C., Morel L. (2020). Immune metabolism regulation of the germinal center response. Exp. Mol. Med..

[39] Waters L.R., Ahsan F.M., Wolf D.M., Shirihai O., Teitell M.A. (2018). Initial B Cell Activation Induces Metabolic Reprogramming and Mitochondrial Remodeling. iScience.

[40] Wilson J.J., Wei J., Daamen A.R., Sears J.D., Bechtel E., Mayberry C.L., Stafford G.A., Bechtold L., Grammer A.C., Lipsky P.E. (2023). Glucose oxidation-dependent survival of activated B cells provides a putative novel therapeutic target for lupus treatment. iScience.

[41] Teng X., Li W., Cornaby C., Morel L. (2019). Immune cell metabolism in autoimmunity. Clin. Exp. Immunol..

[42] Hao Y., Stuart T., Kowalski M.H., Choudhary S., Hoffman P., Hartman A., Srivastava A., Molla G., Madad S., Fernandez-Granda C., Satija R. (2024). Dictionary learning for integrative, multimodal and scalable single-cell analysis. Nat. Biotechnol..

[43] McGinnis C.S., Murrow L.M., Gartner Z.J. (2019). DoubletFinder: Doublet Detection in Single-Cell RNA Sequencing Data Using Artificial Nearest Neighbors. Cell Syst..

[44] Alquicira-Hernandez J., Powell J.E. (2021). Nebulosa recovers single-cell gene expression signals by kernel density estimation. Bioinformatics.

[45] Wolf F.A., Angerer P., Theis F.J. (2018). SCANPY: large-scale single-cell gene expression data analysis. Genome Biol..

[46] Bergen V., Lange M., Peidli S., Wolf F.A., Theis F.J. (2020). Generalizing RNA velocity to transient cell states through dynamical modeling. Nat. Biotechnol..

[47] Weiler P., Lange M., Klein M., Pe'er D., Theis F. (2024). CellRank 2: unified fate mapping in multiview single-cell data. Nat. Methods.

[48] Borcherding N., Vishwakarma A., Voigt A.P., Bellizzi A., Kaplan J., Nepple K., Salem A.K., Jenkins R.W., Zakharia Y., Zhang W. (2021). Mapping the immune environment in clear cell renal carcinoma by single-cell genomics. Commun. Biol..

[49] Popov A., Samokhina M., Balashov I. (2024). immunomind/immunarch: Immunarch 0.9.1.

[50] Gupta N.T., Vander Heiden J.A., Uduman M., Gadala-Maria D., Yaari G., Kleinstein S.H. (2015). Change-O: a toolkit for analyzing large-scale B cell immunoglobulin repertoire sequencing data. Bioinformatics.

[51] Bankhead P., Loughrey M.B., Fernández J.A., Dombrowski Y., McArt D.G., Dunne P.D., McQuaid S., Gray R.T., Murray L.J., Coleman H.G. (2017). QuPath: Open source software for digital pathology image analysis. Sci. Rep..

[52] Stringer C., Wang T., Michaelos M., Pachitariu M. (2021). Cellpose: a generalist algorithm for cellular segmentation. Nat. Methods.

[53] Liu X., Quan N. (2015). Immune Cell Isolation from Mouse Femur Bone Marrow. Bio. Protoc..

[54] Luecken M.D., Theis F.J. (2019). Current best practices in single-cell RNA-seq analysis: a tutorial. Mol. Syst. Biol..

[55] Choudhary S., Satija R. (2022). Comparison and evaluation of statistical error models for scRNA-seq. Genome Biol..

[56] Hao Y., Hao S., Andersen-Nissen E., Mauck W.M., Zheng S., Butler A., Lee M.J., Wilk A.J., Darby C., Zager M. (2021). Integrated analysis of multimodal single-cell data. Cell.

[57] Virshup I., Rybakov S., Theis F.J., Angerer P., Wolf F.A. (2024). anndata: Access and store annotated data matrices. J. Open Source Softw..

[58] Subramanian A., Tamayo P., Mootha V.K., Mukherjee S., Ebert B.L., Gillette M.A., Paulovich A., Pomeroy S.L., Golub T.R., Lander E.S., Mesirov J.P. (2005). Gene set enrichment analysis: a knowledge-based approach for interpreting genome-wide expression profiles. Proc. Natl. Acad. Sci. USA.

[59] Castanza A.S., Recla J.M., Eby D., Thorvaldsdóttir H., Bult C.J., Mesirov J.P. (2023). Extending support for mouse data in the Molecular Signatures Database (MSigDB). Nat. Methods.

