# Supplementary material for: Biotin-dependent metabolism controls germinal center fate and vaccine-induced antibody diversification
Source: iScience. 2026 Oct 21;29(11):117999. doi: 10.1016/j.isci.2026.117999 (PMC13717891; doi:10.1016/j.isci.2026.117999)
Supplement: Supplementary file 1 [file mmc1.pdf]

iScience, Volume *29*

Supplemental information

Biotin-dependent metabolism controls germinal center fate and vaccine-induced antibody diversification

Chu-Han Hsieh, Wilson Aala Jr., Ying-Chen Tsai, Ya-Fang Huang, Miao-Shan Lin, Daw-Yang Hwang, Dar-Bin Shieh, Hsiang-Hsuan Sung, Si-Tse Jiang, Ching-Wei Lin, Chih-An Chen, Michael Warren Hughes, Peng-Chieh Chen, and Chi-Chang Shieh

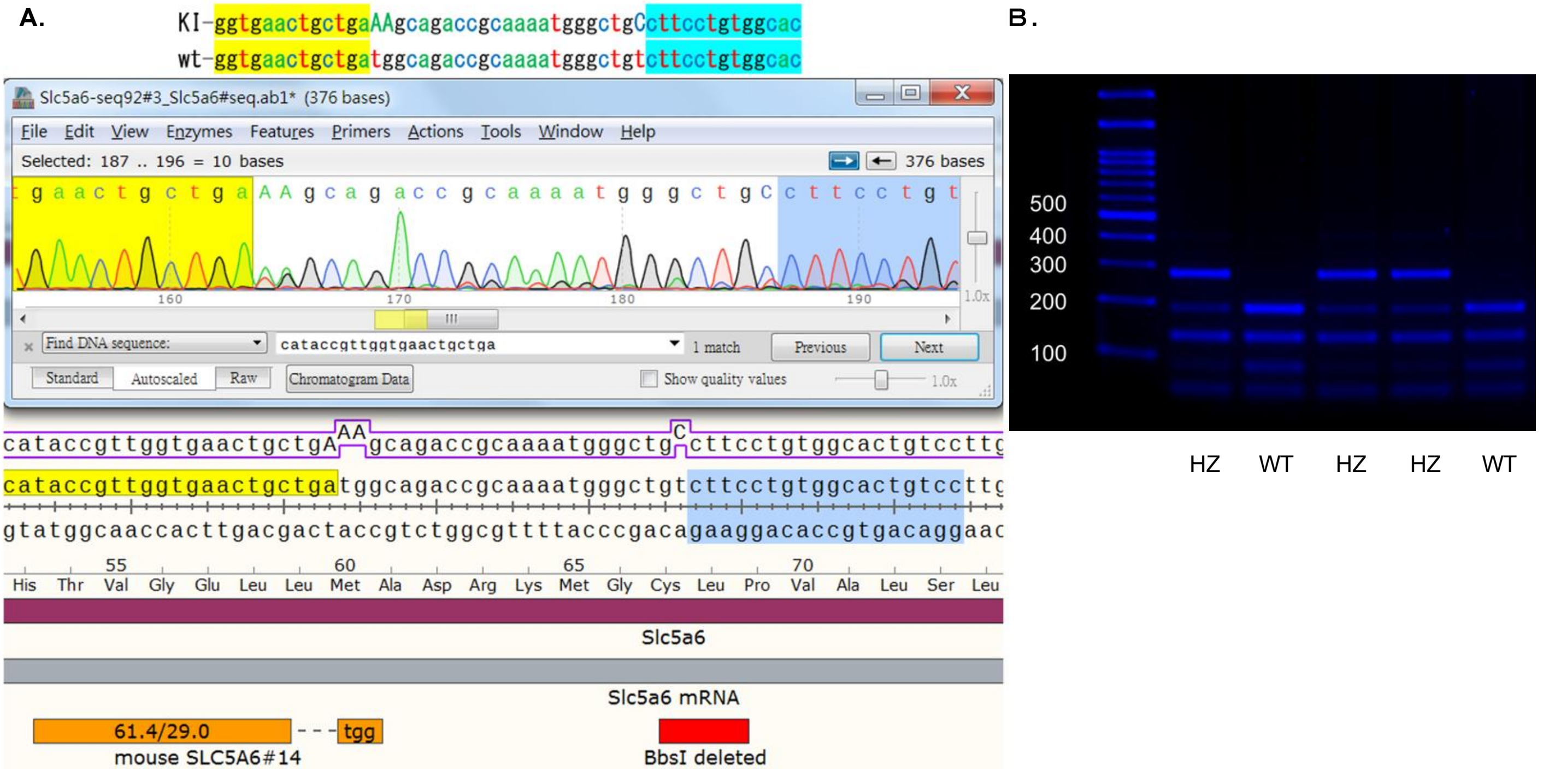

Figure S1 Validation of the *Slc5a6* mutant mouse model and characterization of baseline metabolic and humoral phenotypes. (A) Representative Sanger sequencing chromatograms confirming the *Slc5a6* missense mutation that disrupts the BccI restriction site together with an engineered upstream silent mutation that disrupts the BbsI restriction site. Alignment with the wild-type (WT) sequence highlights the edited nucleotides (yellow and blue shading), and the corresponding amino acid sequence is shown below. The missense mutation introduces the M60K substitution, whereas the upstream silent mutation does not alter the encoded amino acid sequence. (B) Representative PCR genotyping followed by BccI restriction enzyme digestion distinguishing WT and heterozygous (HZ) alleles. WT alleles generate three fragments (186, 133, and 85 bp), whereas HZ mice display both WT- and mutant-derived digestion products. Molecular weight markers (bp) are indicated on the left.

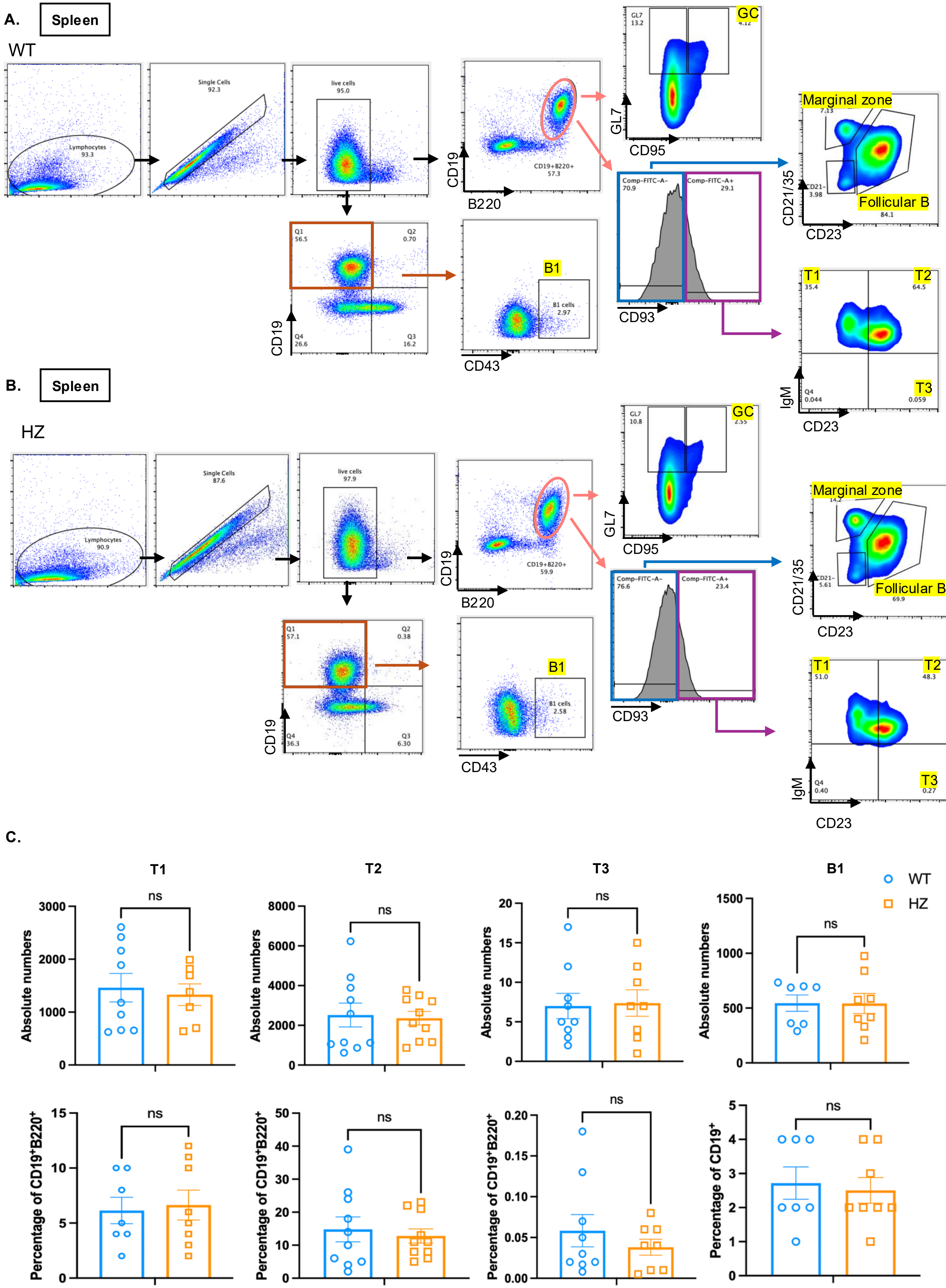
A.
Spleen
WT
Lymphocytes 93.3
Single Cells 92.3
live cells 95.0
CD19+B220+ 57.3
CD19
B220
GL7 13.2
GC 4.12
GL7
CD95
Comp-FITC-A- 70.9
Comp-FITC-A+ 29.1
CD93
Marginal zone 7.13
CD21- 3.98
Follicular B 84.1
CD21/35
CD23
Q1 56.5
Q2 0.70
Q4 26.6
Q3 16.2
B1
B1 cells 2.97
CD43
T1 35.4
T2 64.5
T3 0.059
Q4 0.044
IgM
B.
Spleen
HZ
Lymphocytes 90.9
Single Cells 87.6
live cells 97.9
CD19+B220+ 59.9
GL7 10.8
GC 2.55
Comp-FITC-A- 76.6
Comp-FITC-A+ 23.4
Marginal zone 14.2
CD21- 5.61
Follicular B 69.9
Q1 57.1
Q2 0.38
Q4 36.3
Q3 6.30
B1 cells 2.58
T1 51.0
T2 48.3
T3 0.27
Q4 0.40
C.
T1
T2
T3
B1
WT
HZ
ns
Absolute numbers
Percentage of CD19+B220+
Percentage of CD19+

Figure S2 Flow cytometry gating strategy and quantification of splenic B cell subsets in WT and HZ mice. (A–B) Representative flow cytometry gating strategy for splenic B cell populations from WT (A) and HZ (B) mice. Gating was performed sequentially as follows: lymphocytes → singlets → live cells → $CD19^{+}B220^{+}$ B cells. Germinal center B cells were defined as $CD19^{+}B220^{+}GL7^{+}CD95^{+}$. Follicular B cells were defined as $CD19^{+}B220^{+}CD93^{-}CD21^{+}CD23^{+}$, and marginal zone B cells as $CD19^{+}B220^{+}CD93^{-}CD21^{+}CD23^{-}$. Transitional B cell subsets were defined as T1 ($CD19^{+}B220^{+}CD93^{+}IgM^{+}CD23^{-}$), T2 ($CD19^{+}B220^{+}CD93^{+}IgM^{+}CD23^{+}$), and T3 ($CD19^{+}B220^{+}CD93^{+}IgM^{-}CD23^{+}$). B1 cells were defined as $CD19^{+}CD43^{+}$. (C) Quantification of absolute numbers (top panels) and frequencies (bottom panels) of splenic B cell subsets in WT and HZ mice. Percentages are shown relative to $CD19^{+}B220^{+}$ B cells (T1–T3) or $CD19^{+}$ cells (B1). Data are presented as mean ± SEM with individual biological replicates indicated. Statistical analysis was performed using unpaired two-tailed t-tests; ns, not significant.

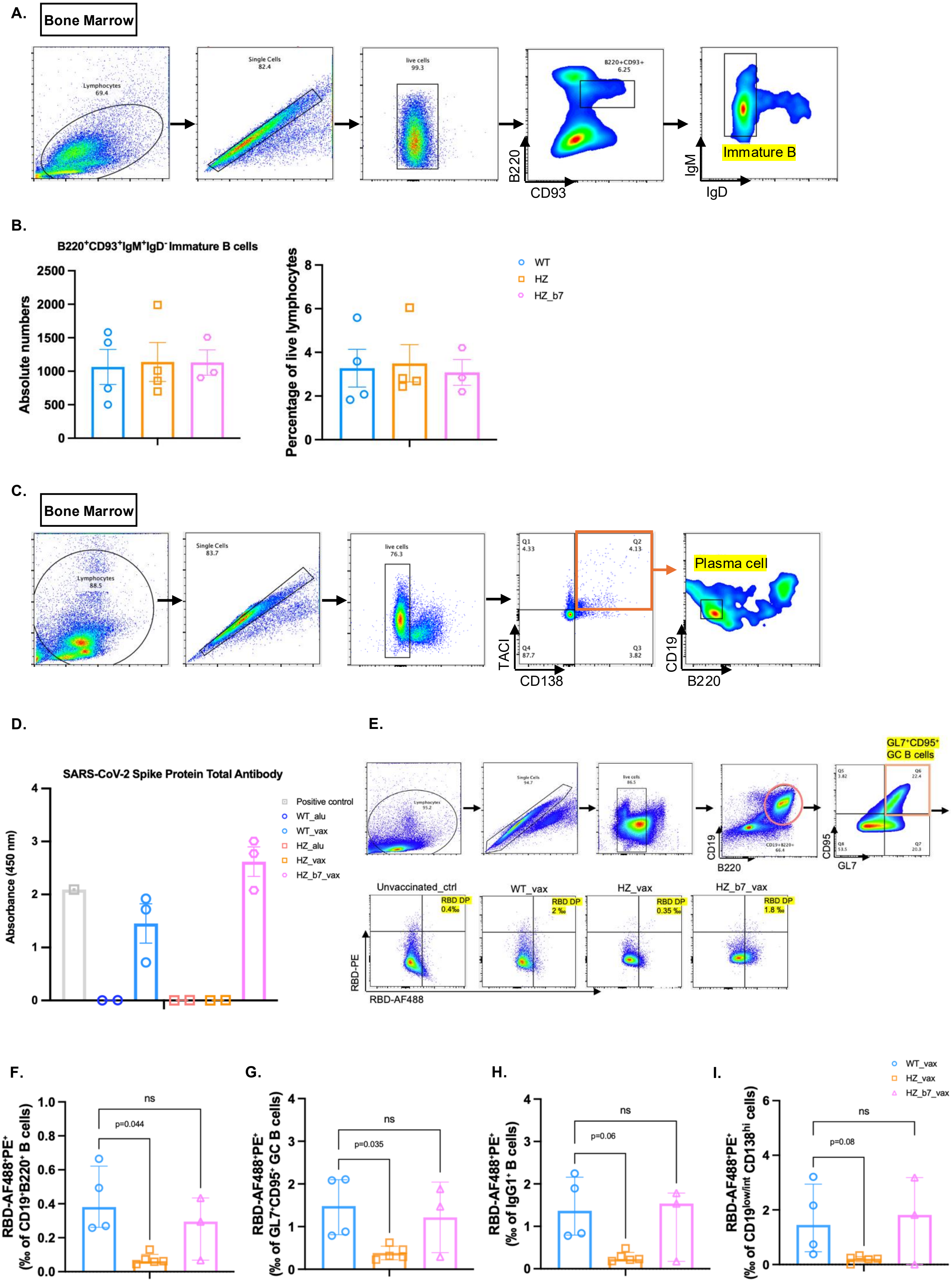
A.
Bone Marrow
Lymphocytes 69.4
Single Cells 82.4
live cells 99.3
B220+CD93+ 6.25
B220
CD93
IgM
IgD
Immature B
B.
B220+CD93+IgM+IgD- Immature B cells
Absolute numbers
Percentage of live lymphocytes
WT
HZ
HZ_b7
C.
Bone Marrow
Lymphocytes 88.5
Single Cells 83.7
live cells 76.3
Q1 4.33
Q2 4.13
Q3 3.82
Q4 87.7
TACI
CD138
Plasma cell
CD19
B220
D.
SARS-CoV-2 Spike Protein Total Antibody
Absorbance (450 nm)
Positive control
WT_alu
WT_vax
HZ_alu
HZ_vax
HZ_b7_vax
E.
GL7+CD95+ GC B cells
CD19
B220
CD95
GL7
Unvaccinated_ctrl
WT_vax
HZ_vax
HZ_b7_vax
RBD DP 0.4‰
RBD DP 2 ‰
RBD DP 0.35 ‰
RBD DP 1.8 ‰
RBD-PE
RBD-AF488
F.
RBD-AF488+PE+ (‰ of CD19+B220+ B cells)
p=0.044
ns
G.
RBD-AF488+PE+ (‰ of GL7+CD95+ GC B cells)
p=0.035
ns
H.
RBD-AF488+PE+ (‰ of IgG1+ B cells)
p=0.06
ns
I.
RBD-AF488+PE+ (‰ of CD19low/int CD138hi cells)
p=0.08
ns
WT_vax
HZ_vax
HZ_b7_vax

Figure S3 Bone marrow B-cell phenotyping and RBD-specific B-cell responses following vaccination. (A) Representative gating strategy for bone marrow immature B cells. Following sequential gating on lymphocytes, singlets, and live cells, immature B cells were identified as $B220^{+}CD93^{+}IgM^{+}IgD^{-}$ immature B cells. (B) Quantification of bone marrow immature B cells. Left: absolute numbers. Right: frequencies among live lymphocytes. No significant differences were observed among WT, HZ, and HZ_b7 mice. (C) Representative gating strategy for bone marrow plasma cells. Following sequential gating on lymphocytes, singlets, and live cells, plasma cells were identified using the previously validated $CD138^{+}TACI^{+}B220^{lo}CD19^{int}$ gating strategy. (D) SARS-CoV-2 spike-specific total antibody responses measured by ELISA. Serum samples collected on day 35 after immunization were analyzed using a commercial SARS-CoV-2 Spike Protein Total Antibody ELISA kit. Antibody levels are presented as OD450 absorbance values. Each symbol represents one mouse, and bars indicate mean ± SEM. Positive control serum supplied with the kit was included as an internal assay control. WT_Alu, WT mice receiving alum alone; WT_Vax, vaccinated WT mice; HZ_Alu, HZ mice receiving alum alone; HZ_Vax, vaccinated HZ mice; HZ_b7_Vax, vaccinated HZ mice receiving biotin supplementation. (E) Representative gating strategy for RBD-specific B cells. Following sequential gating on lymphocytes, singlets, live cells, and $CD19^{+}B220^{+}$ B cells, GC B cells were identified as $GL7^{+}CD95^{+}$ cells. RBD-specific cells were subsequently identified by dual staining with RBD-AF488 and RBD-PE probes. Representative RBD double-positive (RBD-DP) plots are shown for a WT unvaccinated control (Unvaccinated _ctrl), WT_Vax, HZ_Vax, and HZ_b7_Vax mice. The same RBD-DP gate was applied to all samples. (F) Quantification of total RBD-DP B cells in vaccinated mice, expressed as the number of $RBD\text{-}AF488^{+}RBD\text{-}PE^{+}$ cells per 1,000 $CD19^{+}B220^{+}$ B cells (‰). (G) Quantification of RBD-DP GC B cells, expressed as the number of $RBD\text{-}AF488^{+}RBD\text{-}PE^{+}$ cells per 1,000 $GL7^{+}CD95^{+}$ GC B cells (‰). (H) Quantification of RBD-DP $IgG1^{+}$ B cells, expressed as the number of $RBD\text{-}AF488^{+}RBD\text{-}PE^{+}$ cells per 1,000 $IgG1^{+}$ B cells (‰). $IgG1^{+}$ B cells were identified within the $CD19^{+}B220^{+}$ B-cell population. (I) Quantification of RBD-DP cells within the $CD19^{low/int}CD138^{hi}$ population, expressed as $RBD\text{-}AF488^{+}RBD\text{-}PE^{+}$ cells per 1,000 $CD19^{low/int}CD138^{hi}$ cells (‰). $CD19^{low/int}CD138^{hi}$ cells were identified based on CD19 and CD138 expression. For panels F–I, each symbol represents one mouse, with 3–5 biological replicates per group, and data are presented as median with interquartile range. Statistical comparisons were performed using the Kruskal–Wallis test followed by Dunn’s multiple-comparisons test. Exact P values are indicated where shown; ns, not significant.

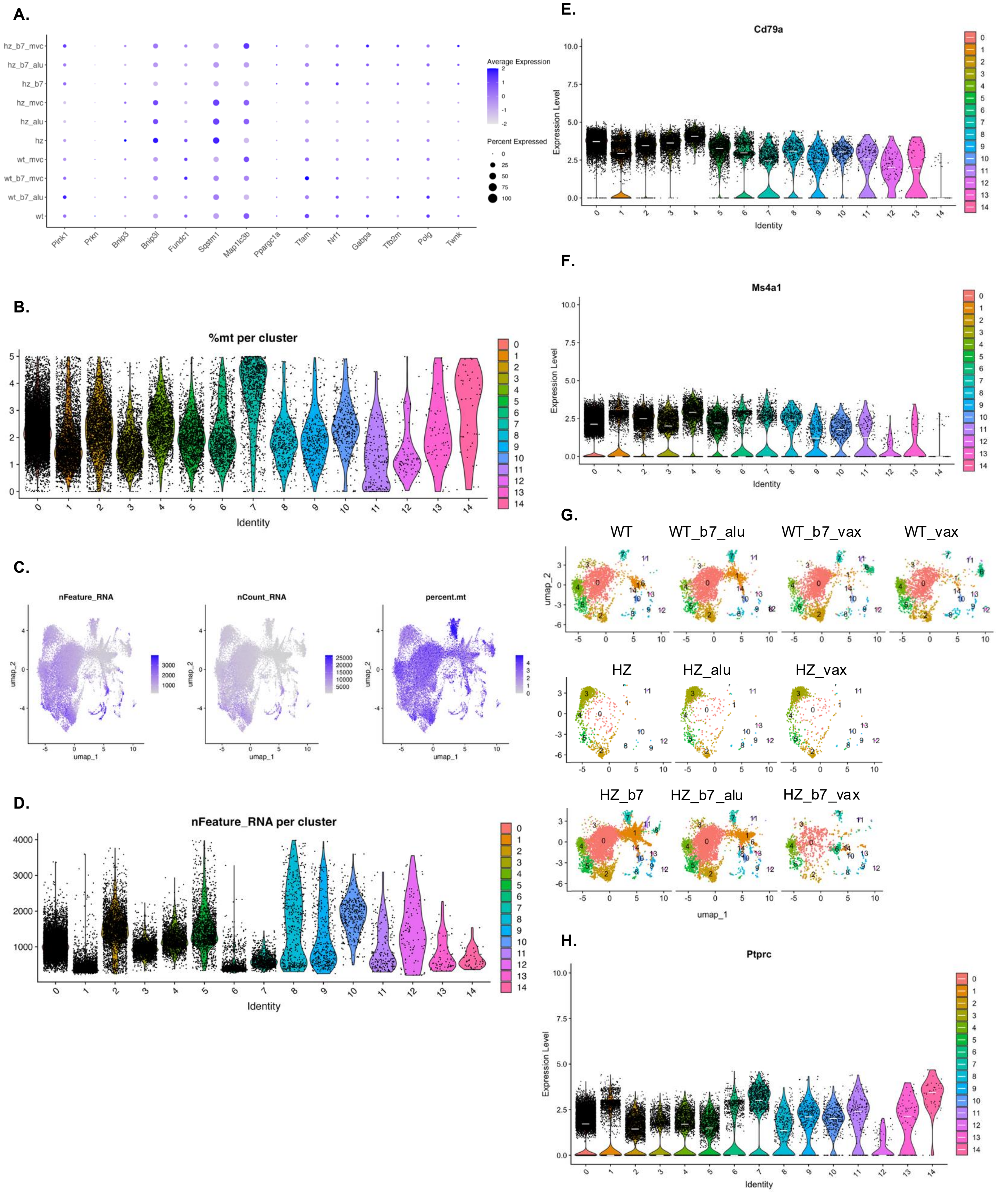

Figure S4 Quality control assessment and marker-based validation of B cell clustering. (A) Dot plot showing normalized expression of representative mitochondrial maintenance and biogenesis-associated genes across experimental groups. (B) Distribution of mitochondrial transcript percentage (percent.mt) across clusters, shown as violin plots. (C) UMAP visualization of quality-control metrics, including the number of detected genes (nFeature_RNA), total UMI counts (nCount_RNA), and mitochondrial transcript percentage (percent.mt). (D) Distribution of detected genes (nFeature_RNA) across clusters. (E,F) Expression of the B-lineage markers *Cd79a* (E) and *Ms4a1* (F) across clusters. (G) UMAP embedding split by genotype. (H) Expression of the pan-leukocyte marker *Ptprc* (CD45) across clusters.

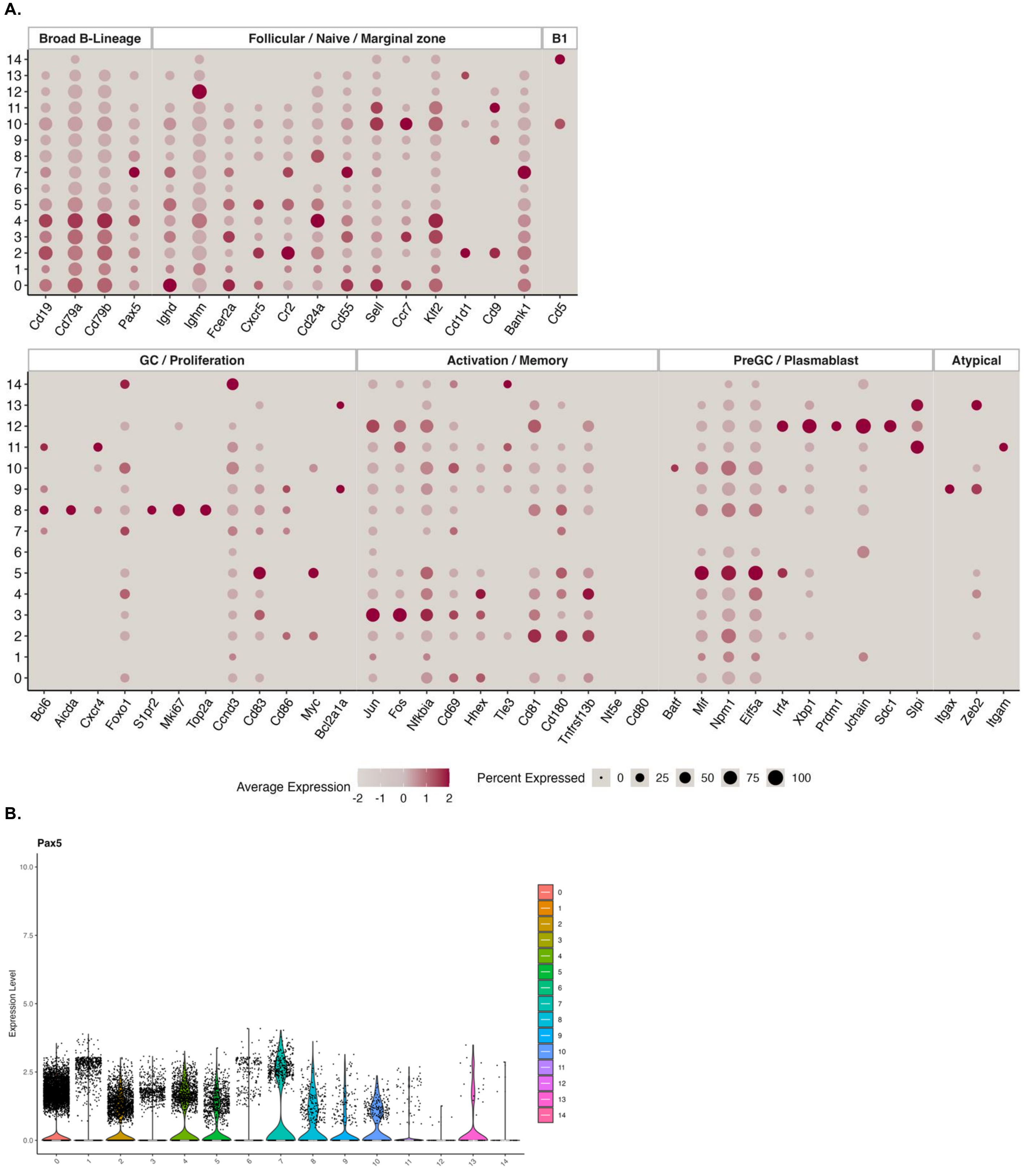

Figure S5 Expanded marker analysis of B-cell cluster annotations. (A) DotPlot showing the expression of canonical B-lineage, follicular, germinal center (GC), activation, proliferation, memory-associated, preGC, plasmablast, and atypical B-cell marker genes across the 15 B-cell clusters identified by unsupervised clustering. Dot size indicates the percentage of cells expressing each gene, and color represents normalized RNA expression values from the Seurat RNA assay after NormalizeData. DotPlots were generated without a minimum expression threshold (dot.min = 0) to provide a complete representation of marker expression across clusters, including low-frequency expression. Cluster annotations were assigned based on the combined expression patterns of multiple canonical markers rather than any single marker gene. (B) Violin plot showing normalized Pax5 expression across all B-cell clusters.

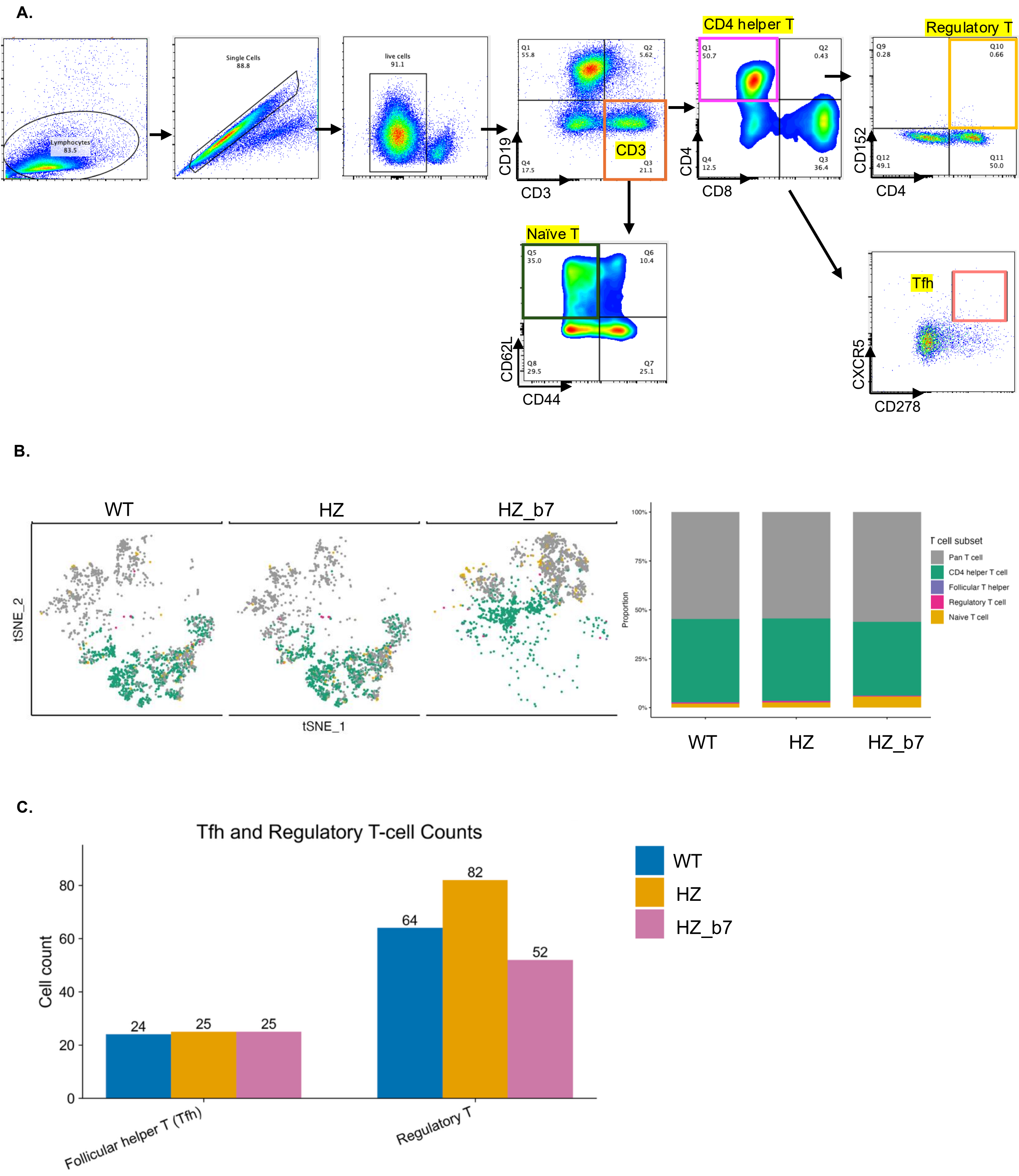

Figure S6 FlowSOM analysis of splenic T-cell populations. (A) Representative flow cytometry dot plots showing the sequential gating strategy for splenic T-cell analysis. Lymphocytes were identified based on forward and side scatter, followed by singlet and live-cell gating. $CD19^{-}CD3^{+}$ T cells were subsequently identified, and major T-cell subsets were defined as follows: CD4 helper T cells ($CD3^{+}CD4^{+}CD8^{-}$), Tfh cells ($CD3^{+}CD4^{+}CXCR5^{+}ICOS/CD278^{+}$), regulatory T cells ($CD3^{+}CD4^{+}CTLA\text{-}4/CD152^{+}$), and naïve T cells ($CD3^{+}CD62L^{+}CD44^{-}$). (B) Shared t-SNE visualization of FlowSOM-clustered splenic T cells from WT, HZ, and HZ_b7 mice (left panel). Relative proportions of the annotated T-cell subsets in WT, HZ, and HZ_b7 mice based on FlowSOM clustering are shown in the right panel. (C) FlowSOM-derived cell counts of Tfh and regulatory T-cell populations across WT, HZ, and HZ_b7 mice.

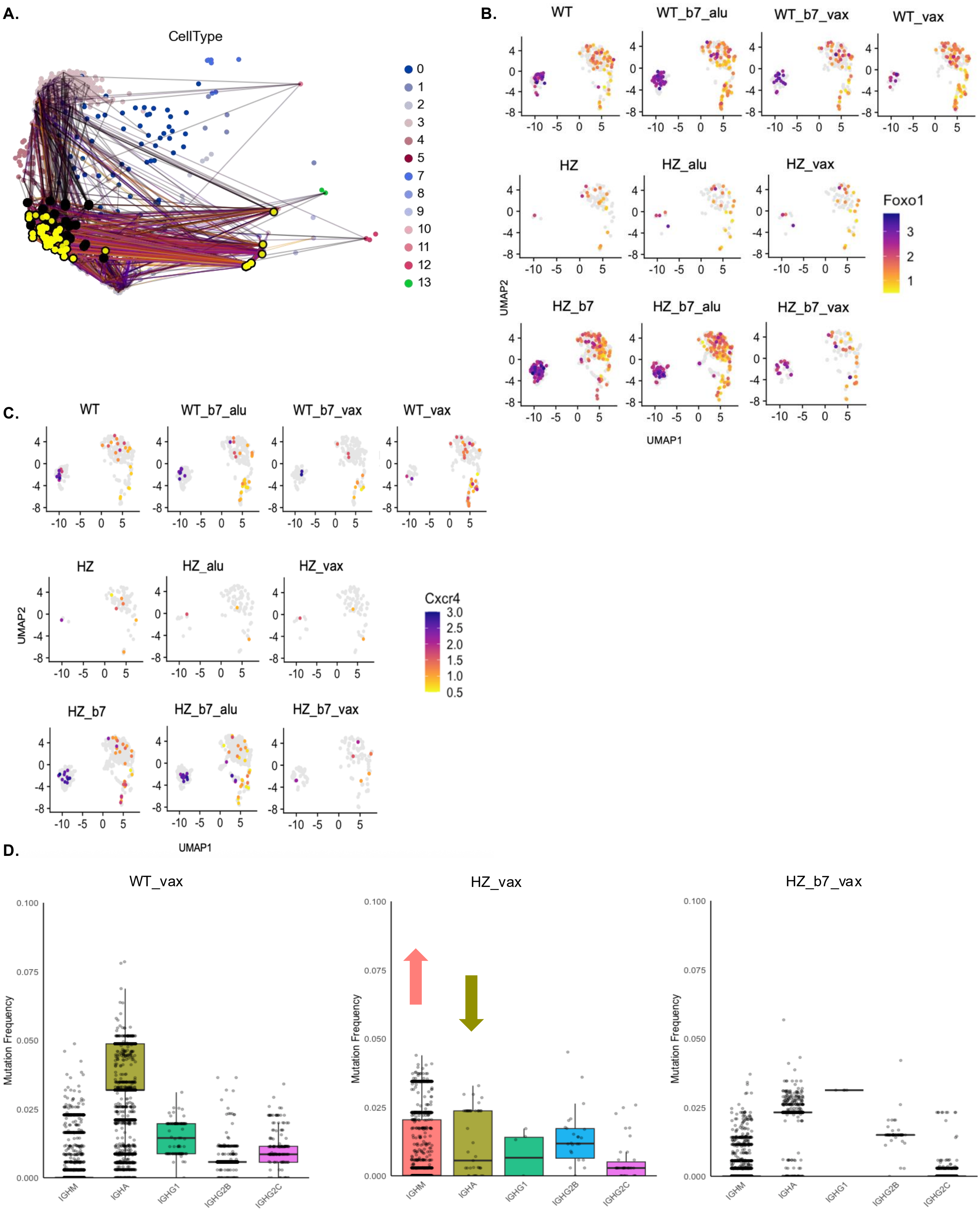

Figure S7 Trajectory analysis, gene expression profiling, and somatic hypermutation across B cell subsets. (A) Random-walk simulations of HZ B cells following vaccination. Black dots indicate cells initialized in the preGC state, and yellow dots denote trajectories that remained stalled within the preGC state. (B) Feature plots showing *Foxo1* expression across UMAP embeddings for each experimental condition. (C) Feature plots of *Cxcr4* expression across experimental groups. (D) Somatic hypermutation (SHM) frequencies in IGHM, IGHA, IGHG1, IGHG2B, and IGHG2C isotypes in WT, HZ, and HZ_b7 mice following vaccination.

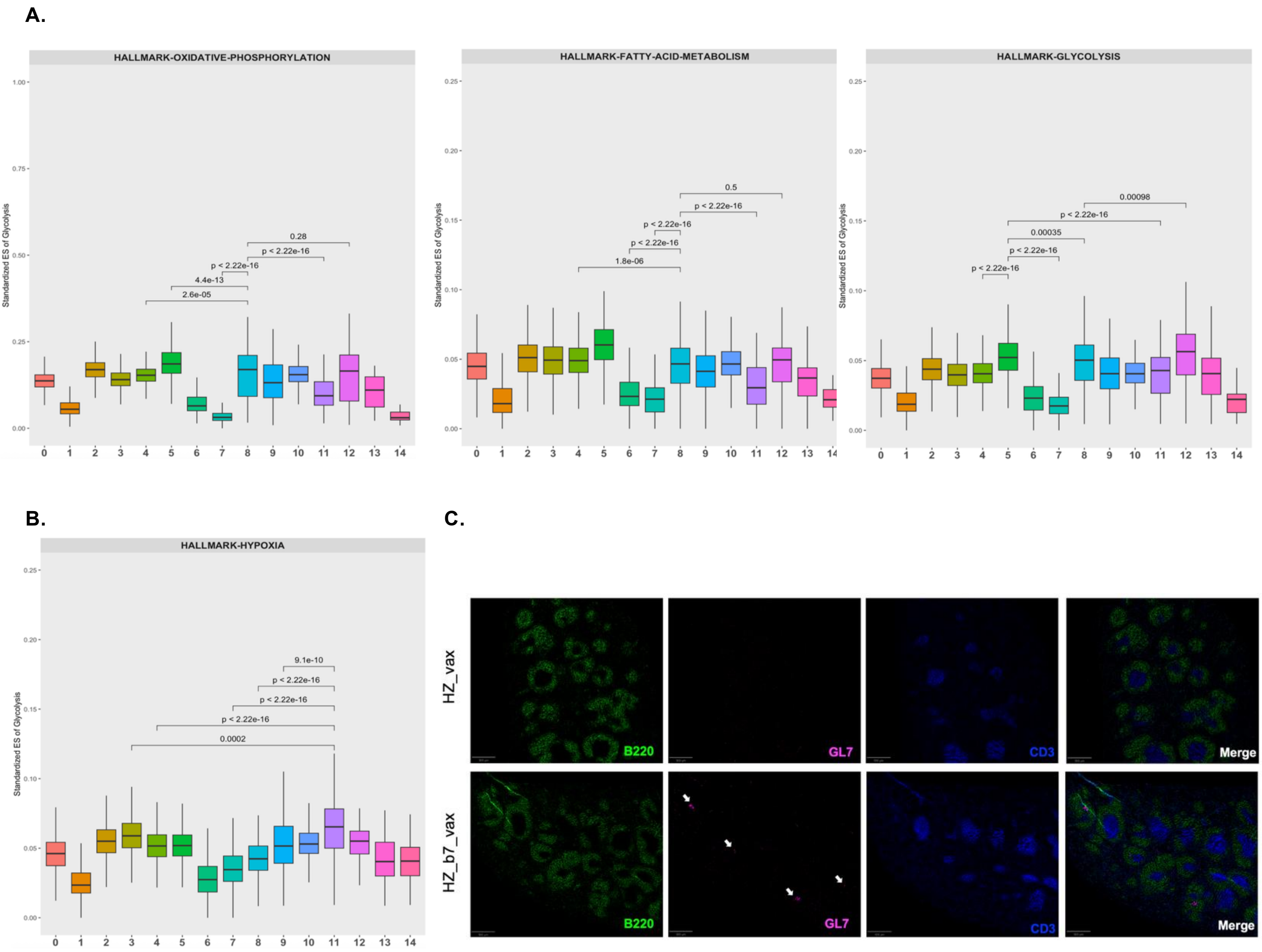

Figure S8 Metabolic pathway enrichment, germinal center marker validation, and low-magnification spleen imaging. (A) Hallmark oxidative phosphorylation, fatty acid metabolism and glycolysis gene set enrichment scores across clusters. (B) Hallmark hypoxia pathway enrichment scores across clusters. (A-B) Statistical comparisons were performed using Wilcoxon rank-sum tests with multiple-testing correction as indicated. (C) Representative low-magnification images of whole spleen sections from HZ_vax and HZ_b7_vax mice stained for B220 (green), GL7 (magenta) (GL7-high regions indicated by white arrow), and CD3 (blue). Images demonstrate overall splenic architecture and distribution of B cell follicles and germinal center-associated signals across conditions. Scale bar: 500 μm.
